# Effects of Exogenous Monosodium Glutamate on GABA Accumulation and Volatile/Nonvolatile Metabolites in Black Highland Barley

**DOI:** 10.1002/fsn3.72125

**Published:** 2026-07-29

**Authors:** Xiaohang Lu, Nana Ma, Yuan Wang

**Affiliations:** ^1^ State Key Laboratory of Plateau Ecology and Agriculture Qinghai University Xining China; ^2^ College of Agriculture and Animal Husbandry Qinghai University Xining China

**Keywords:** γ‐aminobutyric acid, amino acid metabolism, fatty acid profile, metabolomics, monosodium glutamate

## Abstract

γ‐Aminobutyric acid (GABA) is a functional amino acid with multiple health benefits; however, the mechanism of its enrichment in black highland barley, particularly through precursor supplementation, has not been fully elucidated. In this study, the effects of exogenous monosodium glutamate (MSG) on GABA accumulation and metabolite regulation were investigated. Precursor types and germination conditions were optimized using a single‐factor approach, and under the optimal MSG concentration, ultra‐high performance liquid chromatography‐mass spectrometry (UHPLC–MS), gas chromatography–mass spectrometry (GC–MS), and gas chromatography‐ion mobility spectrometry (GC‐IMS) were employed to profile amino acids, fatty acids, and volatile and nonvolatile metabolites. The results showed that MSG addition significantly enhanced GABA accumulation compared with glutamic acid (*p* < 0.05), reaching a maximum of 2.55 ± 0.12 mg·g^−1^. The optimal MSG concentration ranged from 50 to 75 mmol·L^−1^, indicating a concentration‐dependent effect on GABA accumulation. A total of 1248 metabolites were identified, covering multiple classes including amino acids, fatty acids, and volatile compounds, indicating substantial metabolic variation during germination. Mechanistically, MSG was converted into glutamic acid under specific pH conditions and subsequently decarboxylated by glutamate decarboxylase, which was associated with enhanced endogenous GABA accumulation and activation of the GABA shunt. This process may contribute to the supply of carbon skeletons and energy to the tricarboxylic acid cycle, thereby promoting the synthesis of unsaturated fatty acids and amino acid‐derived compounds, and ultimately increasing the levels of flavor‐related metabolites. These findings provide new insights into the regulatory mechanism of GABA enrichment induced by MSG and offer a theoretical basis for improving GABA production and the high‐value utilization of black highland barley.

## Graphical abstract

Overview of MSG‐assisted GABA enrichment and associated changes in amino acid, fatty acid, and volatile metabolite profiles in germinated black highland barley.

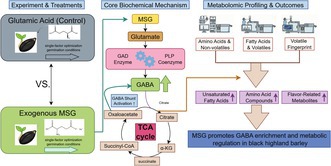

## 1 Introduction

Highland barley ( *Hordeum vulgare* , Poaceae), referred to as Qingke in Chinese and Ne in Tibetan, is a cereal crop primarily cultivated in high‐altitude regions such as the Qinghai‐Tibet Plateau. These regions are characterized by harsh environmental conditions that limit agricultural diversity, thereby increasing dependence on locally adapted, nutrient‐rich staple crops (Obadi et al. 2021). Among the various colored varieties of highland barley—white, blue, purple, and black—the black highland barley (BHB) is particularly esteemed for its high concentrations of amino acids, dietary fiber, anthocyanins, and polyphenols. These compounds are associated with hypoglycemic, hypolipidemic, and antioxidant properties (Chen et al. 2023; Zhang et al. 2021). One important bioactive component of BHB is γ‐aminobutyric acid (GABA), a four‐carbon amino acid that is not part of proteins and is synthesized by the enzyme glutamate decarboxylase (GAD) through the α‐decarboxylation of L‐glutamate. In animals, GABA acts as a main inhibitory neurotransmitter, whereas in plants, it is vital for stress response and managing carbon and nitrogen balance (Tufail et al. 2025). Tibetan highland barley is distinguished by its notably high GABA content, approximately 18.00–28.00 mg/100 g (Bai et al. 2019), surpassing that of other cereals. It has been observed that darker grains tend to accumulate more GABA than lighter ones (Cao et al. 2010). Despite this, the mechanisms underlying GABA enrichment in barley remain insufficiently explored. Current methodologies to enhance GABA accumulation in cereals predominantly involve the supplementation of exogenous precursors such as glutamate or monosodium glutamate (MSG), the application of physical or chemical stressors like anoxia, high salinity, or low temperatures, and the activation of metabolism through germination (Jiang et al. 2021). Germination, in particular, is acknowledged as an effective and practical method, as it reactivates endogenous enzymes such as GAD, increases the flux through the GABA shunt, and does not require specialized inducers (D. Zhang et al. 2021). Considering the suitability of barley for germination‐based processing and its inherent nutritional benefits, this study employs germination as a strategy to further enhance GABA content and to explore the metabolic responses to precursor supplementation.

In cereal grains, such as barley, GABA is integral to stress tolerance, seed germination, and grain quality (Zhou et al. 2021). In plants, glutamate undergoes conversion to GABA via the enzymatic action of GAD, and metabolized through the GABA pathway that connects to the tricarboxylic acid cycle (TCA) (Bhattacharjee et al. 2018). The regulation of plant GAD activity is governed by a compact framework: GAD is activated by the binding of Ca^2+^/calmodulin (CaM) and by cytoplasmic acidification, both of which are induced by germination‐related stimuli such as imbibition, hypoxia, and mechanical stress (Ahmad and Fariduddin 2024; Benidickson et al. 2023). During germination, the reactivation of metabolic processes leads to mild cellular acidosis and transient Ca^2+^ fluxes, which collectively enhance GAD activity and GABA accumulation (Xie et al. 2022). The introduction of exogenous MSG can further influence this system by increasing intracellular glutamate concentrations, thereby shifting the equilibrium of the GAD reaction toward increased GABA production and potentially altering the metabolic flux through the GABA shunt toward succinate and the TCA cycle (Youssef et al. 2023). Synthesized GABA is converted into succinate by the GABA shunt, a metabolic pathway that uses the enzymes GABA transaminase (GABA‐T) and succinic semialdehyde dehydrogenase (SSADH) (Palabıyık et al. 2024). This process facilitates the entry of carbon into the tricarboxylic acid (TCA) cycle, thereby linking nitrogen metabolism with mitochondrial respiration (Bown and Shelp 1997; Fait et al. 2008). In the context of black highland barley, the potential for GABA enrichment is of significant interest due to its possible synergistic interactions with anthocyanins and other phenolic compounds. However, the activity of GAD and the flux through the GABA shunt during germination are not yet well understood. MSG, the sodium salt of glutamic acid, functions as a soluble and nontoxic exogenous precursor of glutamate. When administered during seed soaking and germination, MSG provides an accessible substrate that can be absorbed by the germinating grain and directly converted to GABA via GAD (Jiang et al. 2021). The underlying biochemical rationale is that an increased intracellular concentration of glutamate shifts the GAD reaction toward enhanced GABA production, contingent upon the enzyme's activity and the availability of its cofactor, pyridoxal 5′‐phosphate (Utama et al. 2025). This precursor‐driven approach has been validated in various cereal systems; for instance, supplementation with exogenous glutamate or MSG has been demonstrated to elevate GABA content in germinated rice, buckwheat, and maize, often accompanied by increased GAD activity (Chen et al. 2025; Oh et al. 2019). In barley, preliminary evidence suggests that amino acid feeding can modify nitrogen partitioning; however, systematic investigations into MSG‐induced GABA accumulation and its broader metabolic implications remain limited. Consequently, this study employs MSG as a precursor to glutamate in order to evaluate the hypothesis that the availability of exogenous substrates augments GABA synthesis during the germination of BHB.

The accumulation of GABA during germination has been linked to the enhancement of flavor precursors and nutritional value. The taste quality of sprouted grains is affected by volatile substances like aldehydes, alcohols, ketones, esters, and aromatics, as well as nonvolatile compounds such as amino acids, fatty acids, organic acids, and sugars (Wu et al. 2025). In barley, germination triggers significant metabolic transformations, including the mobilization of storage reserves, activation of lipoxygenase pathways, and production of flavor precursors (Han et al. 2017; Qu et al. 2019). Research on highland barley has revealed varietal differences in volatile profiles; for example, yellow and black varieties exhibit the highest aldehyde content, whereas white barley is abundant in alcohols and blue barley in aromatic compounds (Yin et al. 2022). Wang et al. (2025a, 2025b, 2025) further identified key aroma‐active substances in black highland barley, such as isovaleric acid, (E)‐2‐octenal, vanillin, and γ‐butyrolactone. Nevertheless, significant knowledge gaps remain. The dynamic changes in volatile and nonvolatile metabolites during precursor‐assisted germination have not been systematically characterized using untargeted and targeted metabolomics approaches, while the relationship between GABA accumulation and flavor‐related metabolic pathways, including fatty acid degradation, amino acid catabolism, and Maillard reaction precursors, remains poorly understood. Moreover, the influence of MSG supplementation on the broader metabolic network beyond GABA accumulation has yet to be clarified. Therefore, an integrated metabolomics profiling approach is needed to link precursor‐induced GABA enrichment with broader quality‐related metabolic changes.

Despite the growing interest in enhancing GABA levels in cereal grains, the mechanisms underlying GABA accumulation in black highland barley, particularly in response to precursor supplementation, remain inadequately elucidated. We propose the hypothesis that the application of exogenous MSG augments GABA accumulation during germination by increasing the availability of substrates for glutamate decarboxylation, thereby influencing coordinated changes in GABA‐related metabolic pathways. Furthermore, it is anticipated that MSG treatment will not only elevate GABA content but also induce alterations in amino acid metabolism, fatty acid composition, and volatile compound profiles, indicative of broader metabolic adjustments occurring during germination. Grounded in this hypothesis, the objectives of the present study were as follows: (i) to ascertain the impact of varying MSG concentrations on GABA accumulation during the germination of black highland barley; (ii) to characterize alterations in amino acids, fatty acids, and both volatile and nonvolatile metabolites under optimal conditions; and (iii) to investigate the associations between GABA accumulation and metabolite profiles. The outcomes of this research are intended to contribute to the foundational understanding of GABA enrichment and to facilitate the development of cereal products enriched with GABA.

## 2 Materials and Methods

### 2.1 Materials and Reagents

Black Highland Barley Seeds: Qinghai Hanhe Biotechnology Co. Ltd.; γ‐aminobutyric acid standard: Shanghai Yuanye Biotechnology Co. Ltd.; Sodium tetraborate (analytical grade): Shanghai Yidian Scientific Instruments Co. Ltd.; Phenol, sodium hypochlorite, soluble starch solution (all analytical grade): Tianjin Zhiyuan Chemical Reagent Co. Ltd.

### 2.2 Instruments and Equipment

The experimental equipment is shown in Table 1.

**TABLE 1:** Experiment equipment.

Equipment	Model	Place of production
Electric thermostatic water bath	HH‐6	Shanghai Billon Instrument Co. Ltd. China
Thermostatic oscillator	THZ‐82	Changzhou Guohua Electrical Appliance Co. Ltd., China
Tabletop high‐speed centrifuge	H/T16MN	Hunan Hexi Instrument & Equipment Co. Ltd., China
UV–visible spectrophotometer	UV‐2600	Shimadzu Corporation, Japan
Vortex mixer	Xw‐80A	Haimen Qilinbeier Instrument Manufacturing Co. Ltd., China
Full‐wavelength microplate reader	MULTISKAN Sky	Beijing Pingliyang Economic and Trade Co. Ltd., China.

### 2.3 Methods

#### 2.3.1 Technological Process

Select black highland barley seeds that are plump, undamaged, and in good physiological condition as raw material. Rinse the black highland barley seeds multiple times with distilled water until free of debris and dust, then place them in a cool, well‐ventilated area to dry naturally. Spread the washed and dried black highland barley seeds evenly on a petri dish, arrange them neatly on a clean bench, and sterilize under UV light for 30 min.

Weigh 10 g of the sterilized black highland barley seeds and soak them in a MSG solution of a certain concentration at a specific temperature for a specified duration. Place the cultured black highland barley samples in a −80°C freezer for 1 day, then freeze‐dry them using a vacuum freeze‐dryer. Grind the samples using a laboratory grinder, sieve to obtain powder, and accurately weigh 10 g of the powder. Dissolve it completely in 20 mL of 60% ethanol solution. Centrifuge at 5000 rpm for 3 min. Pour 1 mL of the centrifuged supernatant into a 10 mL test tube for future use. Add the following solutions to the sample solution in sequence: 1 mL of 0.2 mol/L (pH 9.0) H_3_BO_3_‐NaOH buffer solution, 2 mL of 5% phenol solution, and 1 mL of 7% sodium hypochlorite solution. Shake well, heat the test tube in boiling water for 5 min, and then swiftly cool it to room temperature in an ice water bath. After 25 min, measure the absorbance at 645 nm using a spectrophotometer. (Zhu et al. 2024).

#### 2.3.2 Design of Single‐Factor Experiments

Glutamic acid and MSG were selected as candidate culture substrates. Black highland barley seeds were incubated in culture solutions containing 0 mmol/L, 25 mmol/L, 50 mmol/L, 75 mmol/L, 100 mmol/L, and 125 mmol/L of each substrate at 25°C for 6 h. Following the steps outlined in Section 2.3.1, the absorbance of each sample was recorded, and the GABA content was determined using the standard curve. The optimal substrate was determined by comparing the GABA accumulation levels.

Black highland barley seeds were cultured in six groups at 25°C with MSG concentrations of 0 mmol/L, 25 mmol/L, 50 mmol/L, 75 mmol/L, 100 mmol/L, and 125 mmol/L. Sixty milliliters of MSG solution was added to the culture system containing black highland barley seeds and cultured for 6 h, with three replicates per group. GABA content was determined using UV spectrophotometry as described previously. The effect of MSG addition on GABA enrichment was observed to determine the optimal concentration range.

#### 2.3.3 Determination of Amino Acids

Sample extraction: 60 mg samples were taken from −80°C, homogenized with water, then mixed with methanol‐acetonitrile (1:1) and isotopic internal standards, followed by vortexing, low‐temperature sonication, protein precipitation at −20°C, and centrifugation at 4°C to collect supernatants. For chromatographic and mass spectrometric analysis, Agilent 1290 UHPLC was used for separation with gradient elution (mobile phases containing ammonium formate aqueous solution and formic acid‐acetonitrile solution), along with QC samples and standards; SCIEX 6500/5500 QTRAP mass spectrometer was applied for detection in MRM mode, and metabolites were identified via Multiquant 3.0.2 software combined with standards (Zhang et al. 2022).

#### 2.3.4 Determination of Fatty Acids

Sample Extraction: Thaw samples at 4°C, vortex with dichloromethane‐methanol (2:1 v/v), wash with gold‐standard water, dry under nitrogen, reconstitute with hexane + internal standard, methylate, treat again with gold‐standard water, dry under nitrogen, redissolve in hexane, and inject 1 μL (split ratio 10:1) for GC–MS analysis. Chromatography: Separate on HP‐5 ms column with programmed temperature ramping, helium carrier gas, and QC samples. Mass Spectrometry: Agilent 5977B MSD in SCAN/SIM mode with EI source (70 eV); quantify via MSD ChemStation (X. Zhang et al. 2022).

#### 2.3.5 Determination of GABA and GAD Content

Add 0.5 mL of borate buffer (pH 9.0, 0.2 mol/L) and 1 mL of 6% redistilled phenol to 6 test tubes, shake thoroughly. After 2–3 min, introduce 1 mL of 5.2% NaClO solution, shake it well, and then place it in a boiling water bath for 10 min. Quickly move to an ice bath for 20 min, continuously shaking until a blue‐green compound forms. Subsequently, spin at 10,000× g for 5 min to remove impurities, measure the absorbance at 645 nm, and write down the results.

Take 0.5 mL of 50 mmol/L phosphate buffer (pH 5.7) containing 1% MSG and 0.2 mmol/L PLP, add 0.5 mL of the enzyme solution to be tested, and incubate in a 37°C water bath for 2 h. After incubation, place in a boiling water bath for 5 min to inactivate the enzyme. Then add 0.5 mL of borate buffer (pH 9.0, 0.2 mol/L) and 1 mL of 6% redistilled phenol, shake thoroughly. After 2–3 min, add 1 mL of 5.2% NaClO solution, shake thoroughly, then place in a boiling water bath for 10 min. Quickly move to an ice bath for 20 min, continuously shaking until a blue–green compound forms. After extraction, centrifuge at 10,000× g for 5 min to clear impurities, measure the absorbance at 645 nm, and note the results.

#### 2.3.6 Determination of Nontargeted Metabolites

Metabolite extraction: Once the sample has been gradually thawed at 4°C, measure a suitable quantity and mix it with a chilled methanol/acetonitrile/water solution in a 2:2:1 volume ratio. Vortex to combine, carry out low‐temperature ultrasonication for 30 min, and then let it rest at −20°C for 10 min. Centrifuge at 14,000× g at 4°C for 20 min, collect the supernatant, vacuum‐dry it, resuspend in 100 μL of acetonitrile‐water solution (acetonitrile: water = 1:1, v/v) for mass spectrometry analysis, vortex to mix, centrifuge at 14,000× g at 4°C for 15 min, and inject the supernatant for analysis.

Chromatography‐mass spectrometry was performed under the following conditions: Agilent 1290 Infinity LC with a HILIC column, column temperature set at 25°C, flow rate at 0.5 mL/min, and an injection volume of 2 μL. Mobile phase A: water +25 mM ammonium acetate +25 mM ammonia; B: acetonitrile. Gradient elution: 0–0.5 min 95% B; 0.5–7 min B 95% → 65%; 7–8 min 65% → 40%; 8–9 min 40% B; 9–9.1 min 40% → 95%; 9.1–12 min 95% B. Samples stored at 4°C in autosampler, analyzed randomly with QC samples. Mass spectrometry: AB SCIEX Triple TOF 6600; ESI ± modes for primary/secondary spectra. ESI settings: source temp 600°C, Gas1/Gas2 = 60, CUR = 30, ISVF±5500 V. m/z ranges: 60–1000 Da (primary), 25–1000 Da (secondary). Scan accumulation time: 0.20 s/spectrum (primary), 0.05 s/spectrum (secondary). Peak intensity screening, collision energy 35 ± 15 eV.

Data processing: Proteo Wizard was used to convert raw Wiff data into mz XML format, after which XCMS software handled peak alignment, retention time correction, and peak area extraction. The extracted data were sequentially subjected to metabolite structure identification, preprocessing, quality evaluation, and finally data analysis.

#### 2.3.7 Determination of Volatile Substances and Key Volatile Substances

Measure 5.0 g of barley and put it in a 20 mL headspace vial, incubate for 15 min, and inject. Each sample is tested in triplicate. The headspace conditions involve incubation at 60°C at 500 rpm, with a splitless injection of 500 μL and a needle temperature of 85°C.

GC‐IMS Analysis: GC conditions: Column temperature 60°C, high‐purity nitrogen carrier gas (≥ 99.999%), programmed flow rate (2.0 mL/min for 2 min → ramp to 10.0 mL/min over 8 min → 100.0 mL/min over 10 min → 150.0 mL/min over 10 min), runtime 30 min, inlet temperature 80°C. IMS conditions: ^3^H ionization source, 53 mm drift tube (500 V/cm, 45°C), high‐purity nitrogen drift gas (150 mL/min), positive ion mode.

Data Processing: Generate 3D spectra, 2D spectra, difference spectra, fingerprint spectra, and Principal component analysis (PCA) plots of volatile components using VOCAL software (Ge et al. 2025).

#### 2.3.8 Calculating Relative Odor Activity Value (ROAV)

Key volatile flavor compounds in black highland barley with different MSG levels are analyzed by ROAV following the method of Wang et al. (2023).

ROAV is calculated using the following formula (1):

(1) $$\text{ROAV}=100\times \frac{\mathrm{Ci}}{C_{\max}}\times \frac{T_{\max}}{\mathrm{Ti}}$$

where *Ci* and *Ti* denote the relative percentage content and the sensory threshold (μg/kg) for each volatile flavor compound, respectively; *C*_max_ and *T*_max_ indicate the percentage content and the sensory threshold (μg/kg) of the component that most significantly contributes to the sample's overall flavor compounds, respectively.

#### 2.3.9 Data Analysis

After preliminary collation of the experimental data, SPSS 25.0 software was used to analyze amino acids, fatty acids, and untargeted metabolites, and volatile compounds. All data are expressed as mean ± standard deviation (SD), with a statistically significant difference defined as *p* < 0.05. All experiments were performed with 3 technical replicates; untargeted metabolomics detection included 6 biological replicates, and other experiments included 3 biological replicates.

## 3 Results

### 3.1 Effects of MSG and Glutamic Acid on GABA Content in Black Highland Barley

**FIGURE 1:**
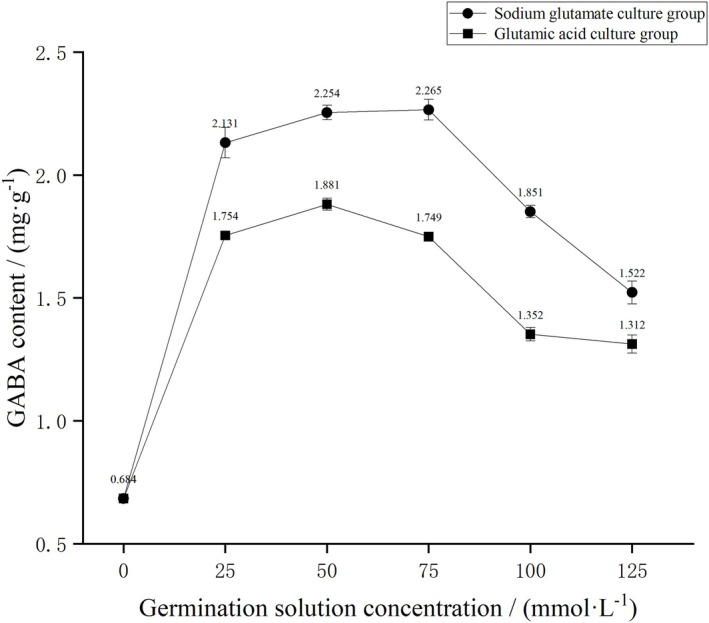
Effects of different germination substrates on GABA content in black highland barley.

As described in Section 2.3.1, the absorbance of the two sample groups was measured to compare GABA content, as shown in Figure 1. The results indicate that the GABA content in black highland barley cultured with MSG as the substrate is significantly higher than that in the group cultured with glutamic acid. Notably, at a germination concentration of 75 mmol/L, the difference in GABA content between the two groups reaches its peak, measuring 2.55 ± 0.12 mg·g^−1^. Consequently, based on these comparative data, MSG was selected as the target substrate for the optimized process evaluation.

As illustrated in Figure 1, the concentration of MSG exerts a significant influence on the GABA content. Within a specific concentration range, the GABA content exhibits a gradual increase corresponding to the rise in MSG concentration. Notably, when the concentration attains the range of 50 to 75 mmol・L^−1^, the GABA content reaches its maximum. However, concentrations exceeding this range result in a decline in GABA content. These findings imply that selecting an optimal MSG concentration, specifically between 50 mmol・L^−1^ and 75 mmol・L^−1^, during the cultivation of black highland barley, can effectively enhance GABA accumulation.

### 3.2 GABA and GAD Content

Drawing upon the findings presented in Section 3.1, MSG concentrations of 0 mmol·L^−1^ (serving as a blank control), 50 mmol·L^−1^, and 75 mmol·L^−1^ were selected for the germination treatment of black highland barley. These experimental groups were designated as Glu‐0, Glu‐50, and Glu‐75, respectively. Subsequent related experiments were then conducted. As illustrated in Table 2, significant differences were observed in the levels of GABA and GAD in black highland barley under the three treatment conditions with varying concentrations of MSG. Notably, the highest concentrations of GABA and GAD were detected in the barley subjected to the 75 mmol·L^−1^ treatment.

**TABLE 2:** Effects of exogenous MSG on GABA and GAD contents in black highland barley.

Parameter	Glu‐0	Glu‐50	Glu‐75	*p*
GABA (mg/g)	0.63 ± 0.06c	2.16 ± 0.16b	2.55 ± 0.12a	< 0.01
GAD (U/g·h)	2.70 ± 0.35c	15.01 ± 2.16b	25.46 ± 3.44a	< 0.01

*Note:* Values are presented as mean ± SD. Different lowercase superscript letters within the same row indicate significant differences among treatments (*p* < 0.05).

### 3.3 Effect of MSG Supplementation on Amino Acid Content in Black Highland Barley

As illustrated in Table 3, a total of 29 amino acid types were identified across the three groups, comprising 9 essential amino acids (EAAs) and 20 nonessential amino acids (NEAAs). The concentration of essential amino acids was found to be highest in the Glu‐0 group and lowest in the Glu‐75 group, with statistical significance (*p* < 0.05). The supplementation of MSG in the Glu‐50 and Glu‐75 groups resulted in a significant increase in the levels of alanine, aminoadipic acid, aspartate, creatine, creatinine, glutamate, hydroxyproline, proline, taurine, valine, and tyrosine (*p* < 0.05). Conversely, the concentrations of bitter amino acids, including arginine, histidine, leucine, isoleucine, phenylalanine, methionine, tryptophan, and lysine, were significantly reduced in the Glu‐50 and Glu‐75 groups compared to the control group (*p* < 0.05). Furthermore, the levels of umami amino acids, such as alanine, aspartate, and glutamate, were significantly elevated in the Glu‐50 and Glu‐75 groups relative to the Glu‐0 group (*p* < 0.05).

**TABLE 3:** Effects of exogenous MSG addition on amino acid content in black highland barley (μmol·L^−1^).

Item	Glu‐0	Glu‐50	Glu‐75
Alanine	1699.20 ± 4.18b	1988.58 ± 13.62a	1719.72 ± 17.80b
Aminoadipic acid	57.37 ± 0.79c	120.28 ± 1.97a	97.56 ± 0.14b
Arginine	225.06 ± 1.50a	9.61 ± 0.18c	113.77 ± 1.37b
Asparagine	440.56 ± 8.58a	391.40 ± 4.67b	184.12 ± 1.81c
Aspartic acid	463.85 ± 4.57c	1269.20 ± 9.77a	946.32 ± 12.20b
Choline	72911.58 ± 530.22a	68199.18 ± 168.93b	66563.37 ± 155.00c
Citrulline	257.67 ± 3.51a	134.57 ± 1.94b	99.65 ± 3.15c
Creatine	0.95 ± 0.03b	1.25 ± 0.05a	0.91 ± 0.02b
Creatinine	0.03 ± 0.00c	0.04 ± 0.00b	0.08 ± 0.00a
Cystine	29.12 ± 0.88a	16.75 ± 0.65b	12.09 ± 0.31c
Glutamate	46711.76 ± 334.88c	239053.17 ± 1457.56b	299976.33 ± 909.14a
Glutamine	784.79 ± 10.33a	271.83 ± 29.38c	619.93 ± 35.01b
Glycine	941.81 ± 1.71a	814.14 ± 6.90b	655.27 ± 8.48c
Histidine	667.12 ± 4.20a	595.27 ± 3.21b	583.93 ± 2.99c
Hydroxyproline	7.79 ± 0.01b	9.65 ± 0.28a	6.65 ± 0.19c
Isoleucine	342.70 ± 11.51a	205.52 ± 1.20b	201.33 ± 0.41b
Leucine	889.18 ± 2.11a	865.13 ± 4.54b	805.37 ± 3.36c
Lysine	819.47 ± 10.38a	731.78 ± 12.08b	398.15 ± 4.90c
Methionine	503.43 ± 17.32a	361.72 ± 12.25b	320.89 ± 6.97c
Ornithine	1692.49 ± 19.59a	1524.71 ± 10.75b	1442.75 ± 16.02c
Phenylalanine	1243.98 ± 47.14a	871.94 ± 1.34b	912.77 ± 11.77b
Proline	464.79 ± 1.87c	590.97 ± 2.56a	502.57 ± 3.52b
Putrescine	44.96 ± 1.07a	7.71 ± 0.13b	8.10 ± 0.04b
Serine	658.83 ± 6.49a	480.16 ± 7.52b	426.61 ± 4.18c
Taurine	3.52 ± 0.03b	3.57 ± 0.01b	3.85 ± 0.03a
Threonine	1224.93 ± 11.31a	654.27 ± 10.11c	776.85 ± 16.89b
Tryptophan	185.97 ± 4.18a	162.41 ± 1.39b	141.49 ± 0.37c
Tyrosine	72.65 ± 0.35c	107.74 ± 0.57b	438.99 ± 6.68a
Valine	1492.20 ± 9.54c	1993.01 ± 9.64a	1784.65 ± 4.68b
EAA	7368.99 ± 75.47a	6441.05 ± 39.87b	5925.43 ± 53.26c
NEAA	127468.77 ± 1028.72c	314994.53 ± 2030.00b	373818.64 ± 1016.74a
TAA	134837.76 ± 1094.22c	321435.58 ± 2063.51b	379744.08 ± 1015.44a
UAA	50159.32 ± 356.85c	242636.75 ± 1489.05b	303498.97 ± 948.03a
BAA	5938.33 ± 90.91a	5542.41 ± 34.15b	5380.45 ± 36.53c

*Note:* Values are presented as mean ± SD. Different lowercase superscript letters within the same row indicate significant differences among treatments (*p* < 0.05).

Abbreviations: BAA, bitter amino acids; EAA, essential amino acids; NEAA, nonessential amino acids; TAA, total amino acids; UAA, umami amino acids.

### 3.4 Effect of MSG Supplementation on Fatty Acid Content in Black Highland Barley

Table 4 shows that black highland barley contains 38 different fatty acids, including 16 saturated, 8 monounsaturated, and 14 polyunsaturated fatty acids. Palmitic acid and stearic acid were the main saturated fatty acids, with their levels significantly higher in the Glu‐50 and Glu‐75 groups than in the Glu‐0 group (*p* < 0.05). Regarding the unsaturated fatty acids, palmitoleic acid, oleic acid, and eicosenoic acid were the most abundant, with their levels increasing in correlation with the amount of MSG supplementation. Linoleic acid and linolenic acid were the most abundant polyunsaturated fatty acids, with their highest levels found in the Glu‐50 group. In addition, the Glu‐50 and Glu‐75 groups showed significantly higher concentrations of eicosapentaenoic acid (EPA) and docosahexaenoic acid (DHA), essential polyunsaturated fatty acids for human health, than the Glu‐0 group (*p* < 0.05).

**TABLE 4:** Effects of exogenous MSG on fatty acid contents in black highland barley (μg·mL^−1^).

Item	Glu‐0	Glu‐50	Glu‐75
C6:0	0.02 ± 0.00c	0.02 ± 0.00b	0.02 ± 0.00a
C8:0	0.17 ± 0.00c	0.21 ± 0.00b	0.29 ± 0.00a
C10:0	0.11 ± 0.00c	0.11 ± 0.00b	0.15 ± 0.00a
C11:0	0.03 ± 0.00b	0.03 ± 0.00b	0.03 ± 0.00a
C12:0	0.90 ± 0.00b	0.87 ± 0.00c	0.93 ± 0.00a
C13:0	0.04 ± 0.00b	0.05 ± 0.00a	0.05 ± 0.00a
C14:0	13.28 ± 0.03c	13.51 ± 0.05b	15.48 ± 0.04a
C15:0	4.50 ± 0.01c	5.25 ± 0.01b	5.39 ± 0.01a
C16:0	1185.42 ± 4.34c	1439.66 ± 4.83a	1339.85 ± 0.95b
C17:0	4.74 ± 0.02c	5.51 ± 0.02b	5.79 ± 0.02a
C18:0	103.63 ± 0.34c	117.70 ± 0.38a	116.35 ± 0.03b
C20:0	14.52 ± 0.02b	16.11 ± 0.06a	16.21 ± 0.10a
C21:0	0.94 ± 0.00c	1.33 ± 0.00a	1.15 ± 0.00b
C22:0	3.49 ± 0.03a	3.15 ± 0.02b	2.99 ± 0.01c
C23:0	1.67 ± 0.00c	2.13 ± 0.01a	1.94 ± 0.01b
C24:0	7.68 ± 0.03b	7.59 ± 0.04b	8.30 ± 0.04a
Sfa	1341.14 ± 4.82c	1613.23 ± 5.42a	1514.92 ± 1.21b
C14:1n5	0.22 ± 0.00c	0.27 ± 0.00b	0.28 ± 0.00a
C16:1n7	12.21 ± 0.00c	14.38 ± 0.01a	13.75 ± 0.03b
C17:1n7	6.46 ± 0.04a	2.86 ± 0.04c	3.57 ± 0.02b
C18:1tn9	0.22 ± 0.00a	0.11 ± 0.00c	0.16 ± 0.00b
C18:1n9	912.77 ± 1.15c	1195.87 ± 1.75a	1077.31 ± 0.51b
C20:1n9	49.62 ± 0.15c	73.97 ± 0.08a	55.74 ± 0.17b
C22:1n9	7.68 ± 0.03c	11.73 ± 0.01a	8.33 ± 0.04b
C24:1n9	8.61 ± 0.03c	14.65 ± 0.02a	8.89 ± 0.02b
Mufa	997.80 ± 1.23c	1313.85 ± 1.80a	1168.04 ± 0.40b
C18:2ttn6	0.60 ± 0.00a	0.55 ± 0.00b	0.50 ± 0.00c
C18:2n6	2361.01 ± 2.81c	2898.37 ± 1.76a	2691.66 ± 4.74b
C18:3n3	330.52 ± 0.88c	543.11 ± 2.34a	379.13 ± 0.99b
C18:3n6	5.25 ± 0.03a	5.27 ± 0.02a	5.12 ± 0.05b
C20:2n6	5.58 ± 0.01c	7.48 ± 0.03a	6.73 ± 0.02b
C20:3n3	0.28 ± 0.00c	0.46 ± 0.00a	0.32 ± 0.00b
C20:3n6	1.25 ± 0.00a	0.96 ± 0.00c	0.98 ± 0.00b
C20:4n6 (AA)	0.17 ± 0.01a	0.08 ± 0.00b	0.07 ± 0.00b
C20:5n3 (EPA)	5.62 ± 0.01c	6.18 ± 0.02a	6.01 ± 0.02b
C22:2n6	0.45 ± 0.00c	0.61 ± 0.00a	0.55 ± 0.00b
C22:4n6	0.47 ± 0.00a	0.41 ± 0.00b	0.39 ± 0.00c
C22:5n3	0.13 ± 0.00b	0.08 ± 0.00c	0.15 ± 0.00a
C22:5n6	9.15 ± 0.04b	9.91 ± 0.03a	9.20 ± 0.04b
C22:6n3 (DHA)	4.53 ± 0.04c	7.60 ± 0.01a	4.97 ± 0.02b
PUFA	2725.02 ± 3.26c	3481.07 ± 2.71a	3105.77 ± 3.67b
Total‐n3	341.07 ± 0.90c	557.44 ± 2.36a	390.58 ± 1.01b
Total‐n6	2383.94 ± 2.89c	2923.63 ± 1.74a	2715.19 ± 4.65b

*Note:* Values are presented as mean ± SD. Different lowercase superscript letters within the same row indicate significant differences among treatments (*p* < 0.05).

### 3.5 Effects of MSG Supplementation on Untargeted Metabolites in Black Highland Barley

#### 3.5.1 Evaluation of the Quality of Experimental Data

Figure S1 shows an overlay comparison of the total ion chromatograms (TICs) for the quality control (QC) samples. The consistency in response intensities and retention times for each chromatographic peak indicates that instrument error had minimal impact during the experiment.

**FIGURE 2:**
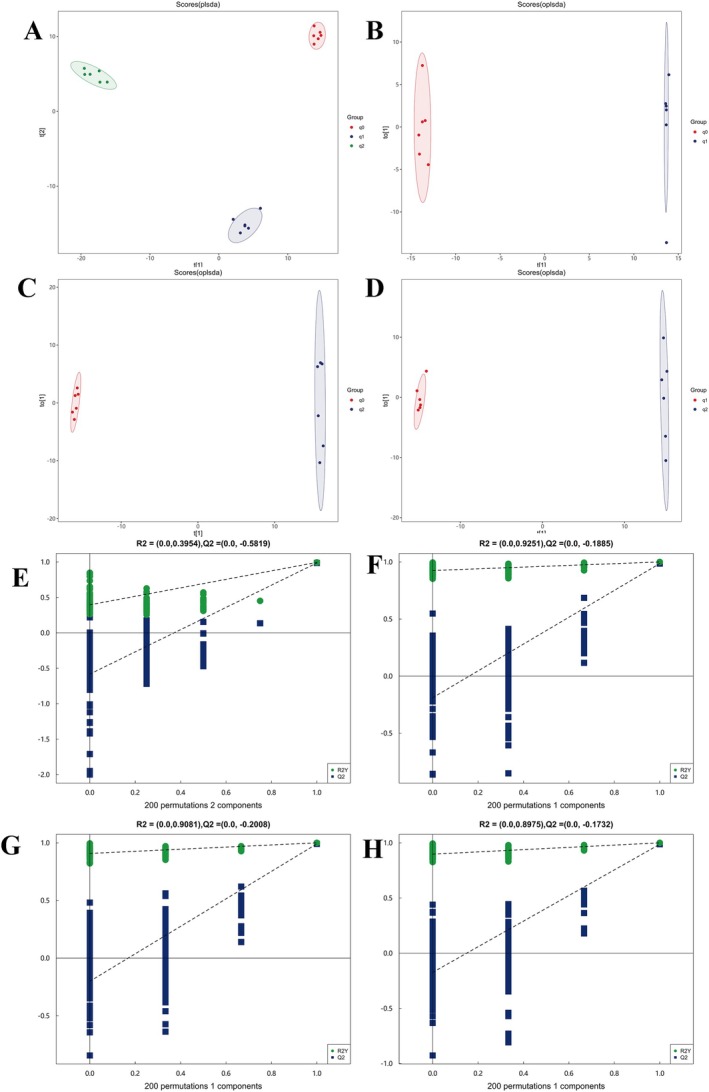
Multivariate statistical analysis of black highland barley cultured with different concentrations of exogenous MSG: PLS‐DA (A), OPLS‐DA (B, Glu‐0 vs. Glu‐50), OPLS‐DA (C, Glu‐0 vs. Glu‐75), OPLS‐DA (D, Glu‐50 vs. Glu‐75), scores of all samples in positive ion mode, permutation test of PLS‐DA (E), and permutation tests of OPLS‐DA (F–H) in positive ion detection mode.

To enhance the discriminatory capacity among the groups, this research employed PLS‐DA and OPLS‐DA to compare the three sample groups after excluding QC samples. As illustrated in Figure 2, both analytical methods effectively distinguished samples from different groups under positive ion mode. A permutation test was performed to determine if the models were overfitting, thereby assessing their reliability. The PLS‐DA and OPLS‐DA score plots showed clear intragroup clustering and intergroup separation among the treatments (Figure 2A–D). The corresponding permutation tests (Figure 2E–H) indicated that the models were reliable and showed no obvious overfitting. The results of the permutation test demonstrated that the differences in metabolites among the groups, as analyzed by PLS‐DA and OPLS‐DA, were statistically significant, exhibiting distinct intergroup variations and robust model stability. The Glu‐75 and Glu‐0 groups exhibited the highest Q^2^ value (0.991), indicating that the metabolic perturbation induced by the Glu‐75 treatment was the most pronounced. Although the R^2^X value for the Glu‐50 versus Glu‐75 comparison was comparable to those observed in the other group comparisons, the Q^2^ value was slightly lower. This suggests that, despite the significant differences between the Glu‐50 and Glu‐75 groups, their patterns of metabolic change may be more similar.

#### 3.5.2 Bioinformatics Analysis of Differential Metabolites

In this study, a comprehensive analysis identified 1248 metabolites in the positive ion mode and 768 metabolites in the negative ion mode. Due to the superior metabolite coverage observed in the positive ion mode, subsequent analyses were conducted using data from this mode. Utilizing the criteria for differential metabolite identification (VIP > 1 and *p* < 0.05), a total of 1103 significantly altered metabolites were discerned across the three sample groups in the positive ion mode. Of these, 649 metabolites were successfully mapped to the KEGG metabolic pathway database.

**FIGURE 3:**
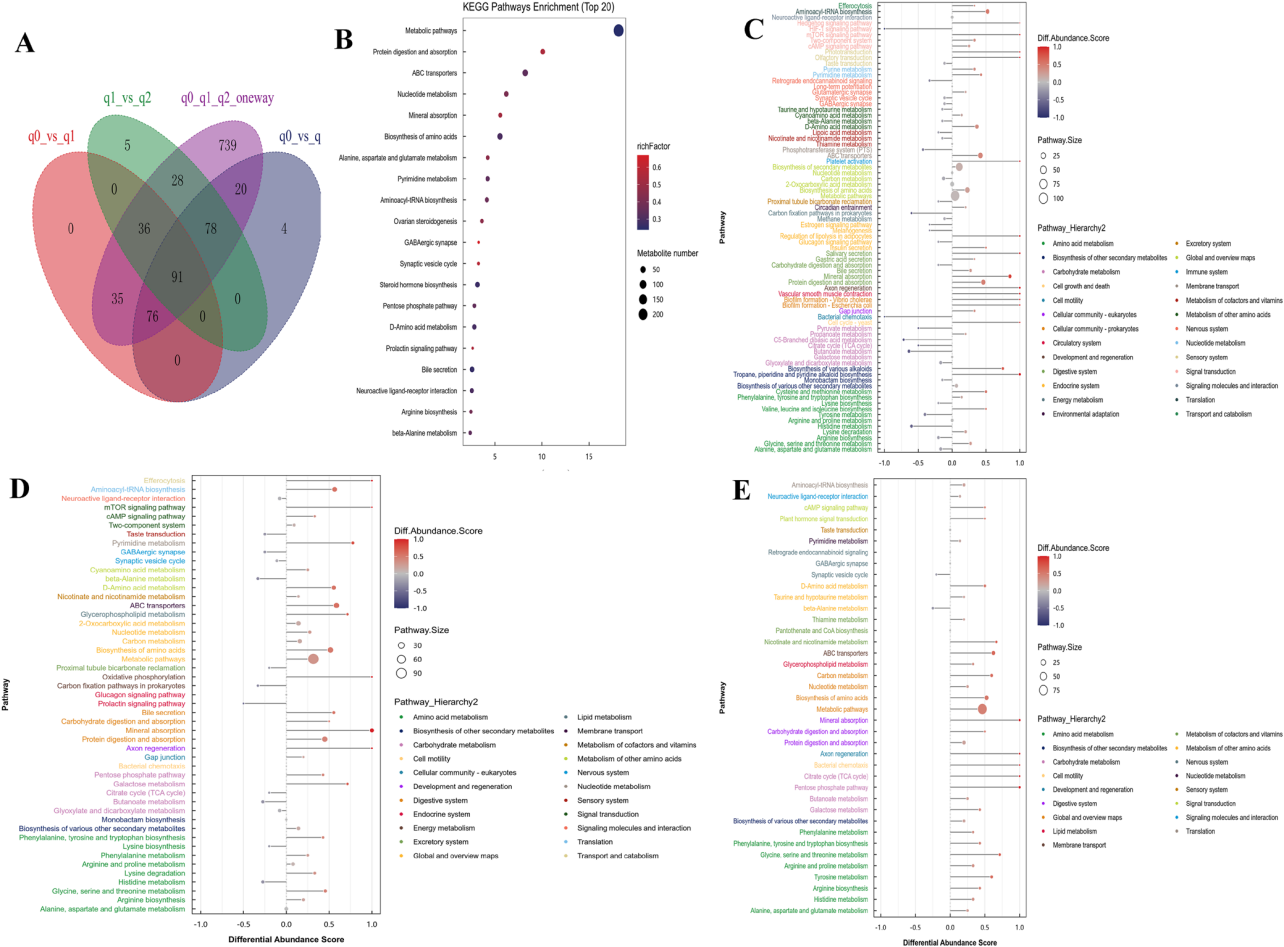
(A) Venn diagram illustrating the overlap of different group‐associated DFMs among the three groups in Black Highland barley. (B) Top 20 enriched KEGG pathways from the comparison among the three groups. Differential abundance score maps of differential metabolic pathways: (C) Glu‐0 vs. Glu‐50, (D) Glu‐0 vs. Glu‐75, (E) Glu‐50 vs. Glu‐75. A score of 1 indicates that the expression of all identified metabolites in the pathway is up‐regulated, and −1 indicates that the abundance of all identified metabolites in the pathway is down‐regulated.

As illustrated in Figure 3A, the comparative analysis identified 238 differential metabolites between the Glu‐0 and Glu‐50 groups, 269 between the Glu‐0 and Glu‐75 groups, and 238 between the Glu‐50 and Glu‐75 groups. In total, 1103 differential metabolites were discerned across all three groups. Comprehensive lists of these metabolites are available in Table S1. Figure 3B presents the KEGG pathway enrichment bubble plot analysis, highlighting the top 20 pathways enriched in each group of black hulless barley subjected to varying concentrations of exogenous MSG. The majority of these pathways are associated with amino acid metabolism, lipid metabolism, and nucleotide metabolism. To facilitate a comparative analysis of the differential metabolic pathways engaged by the differential metabolites in black highland barley under different MSG concentrations, differential enrichment score plots were generated (Figure 3C–E).

As shown in Table 5, the Glu‐0 group displayed seven up‐regulated and five down‐regulated metabolic pathways in contrast to the Glu‐50 group, with a differential abundance score surpassing 0.5 and a significance level of *p* < 0.05. The up‐regulated pathways comprised Aminoacyl‐tRNA biosynthesis, Mineral absorption, Valine, leucine, and isoleucine biosynthesis, Regulation of lipolysis in adipocytes, Cysteine and methionine metabolism, Tropane, piperidine, and pyridine alkaloid biosynthesis, and the Biosynthesis of various alkaloids. Conversely, the down‐regulated pathways included Butanoate metabolism, Histidine metabolism, the Citrate cycle (TCA cycle), and Pyruvate metabolism. The key metabolites identified were Pyruvate, DL‐threonine, Leucine, Phenylalanine, Tryptophan, DL‐tryptophan, Alanine, L‐Alanine, DL‐serine, Glycine, L‐methionine, D‐glutamine, DL‐proline, L‐aspartic acid, Alpha‐ketoglutarate, DL‐Glutamic acid, Glutamic acid, L‐Glutamate, Succinate, γ‐aminobutyric acid/4‐Aminobutyric acid, and Adenosine 3′,5′‐cyclic monophosphate.

**TABLE 5:** Differential metabolites in key enriched KEGG pathways of black highland barley (*p* < 0.05).

Metabolic pathways	Metabolites
Glu‐0 vs. Glu‐50	
Upregulation in the Glu‐0 group	
Aminoacyl‐tRNA biosynthesis	Alanine, DL‐Glutamic acid, DL‐serine, DL‐threonine, DL‐tyrosine, Glutamic acid, Glycine, L‐Alanine, L‐aspartic acid, L‐methionine, Leucine, Lysine, Phenylalanine, Tryptophan, Arginine, D‐glutamine, DL‐proline, DssL‐tryptophan, Histidine, L‐Glutamate, Tyrosine
Mineral absorption	Alanine, D‐ (+)‐galactose, DL‐serine, DL‐threonine, Glycine, L‐Alanine, L‐methionine, Leucine, Phenylalanine, Phosphoric acid, Tryptophan, D‐glutamine, DL‐proline, DL‐tryptophan
Valine, leucine and isoleucine biosynthesis	DL‐threonine, Ketoleucine, Leucine, Pyruvate
Regulation of lipolysis in adipocytes	Adenosine 3′,5′‐cyclic monophosphate, Corticosterone, Cyclic gmp
Cysteine and methionine metabolism	Alanine, DL‐serine, L‐Alanine, L‐aspartic acid, L‐glutathione, reduced, L‐methionine, Pyruvate, Methionine sulfoxide
Tropane, piperidine and pyridine alkaloid biosynthesis	Lysine, Nicotinate, Phenylalanine, Phenyllactic acid, L‐pipecolic acid, Scopolamine, Trigonelline
Biosynthesis of various alkaloids	Cinchonine, Phenylalanine, Salicylic acid, Tryptophan, 2‐methylpyrrolidine, Cucurbitacin b, Cucurbitacin d, DL‐tryptophan
Downregulation in the Glu‐0 group	
Butanoate metabolism	γ‐aminobutyric acid, γ‐hydroxybutyric acid, 4‐Aminobutyric acid (GABA), Alpha‐ketoglutarate, Butanoic acid, DL‐Glutamic acid, Glutamic acid, Pyruvate, Succinate, Diacetyl, L‐Glutamate
Histidine metabolism	Alpha‐ketoglutarate, DL‐Glutamic acid, Glutamic acid, L‐aspartic acid, L‐Carnosine, L‐Histidinol phosphate, Histamine, Histidine, L‐Glutamate, N‐acetylhistamine
Citrate cycle (TCA cycle)	Alpha‐ketoglutarate, Cis‐aconitate, Pyruvate, Succinate
Pyruvate metabolism	L‐(+)‐lactic acid, Pyruvaldehyde, Pyruvate, Succinate
Glu‐0 vs. Glu‐75	
Upregulation in the Glu‐0 group	
Carbohydrate digestion and absorption	Butanoic acid, D‐(+)‐galactose, Propionic acid, D‐glucose 6‐phosphate
Biosynthesis of amino acids	2‐Oxoadipic acid, 3‐dehydroshikimate, Alanine, Alpha‐ketoglutarate, Citrate, Dihydroxyacetone phosphate, DL‐Glutamic acid, DL‐serine, DL‐threonine, DL‐tyrosine, Glutamic acid, Glycine, Ketoleucine, L‐Alanine, L‐aspartic acid, L‐methionine, Leucine, Lysine, N‐α‐acetyl‐l‐ornithine, Phenylalanine, Tryptophan, 2‐aminoadipic acid, Arginine, D‐erythro‐imidazolylglycerol phosphate, D‐glutamine, DL‐asparagine, DL‐proline, DL‐tryptophan, Histidine, L‐citrulline, L‐Glutamate, L‐Proline, Tyrosine
D‐Amino acid metabolism	Alanine, Alpha‐ketoglutarate, DL‐Glutamic acid, DL‐serine, DL‐threonine, Glutamic acid, Glycine, L‐Alanine, L‐aspartic acid, L‐methionine, Lysine, Phenylalanine, Arginine, D‐glutamine, DL‐proline, Histidine, L‐Glutamate, L‐Proline
Aminoacyl‐tRNA biosynthesis	Alanine, DL‐Glutamic acid, DL‐serine, DL‐threonine, DL‐tyrosine, Glutamic acid, Glycine, L‐Alanine, L‐aspartic acid, L‐methionine, Leucine, Lysine, Phenylalanine, Tryptophan, Arginine, D‐glutamine, DL‐asparagine, DL‐proline, DL‐tryptophan, Histidine, L‐Glutamate, L‐Proline, Tyrosine
ABC transporters	Alanine, Cytidine, D‐mannitol, D‐Mannose, Deoxyinosine, DL‐Glutamic acid, DL‐serine, DL‐threonine, Glutamic acid, Glycine, His‐Lys, Inosine, L‐Alanine, L‐aspartic acid, Leucine, Lysine, Myo‐inositol, Phenylalanine, Phosphoric acid, Uridine, Arginine, Betaine, Choline, Ciprofloxacin, D‐glutamine, DL‐proline, Histidine, L‐Glutamate, L‐Proline
Galactose metabolism	α‐d‐galactose 1‐phosphate, 2‐dehydro‐3‐deoxy‐d‐gluconate, D‐(+)‐galactose, D‐Mannose, Dihydroxyacetone phosphate, Galactinol, Myo‐inositol
Glycerophospholipid metabolism	Dihydroxyacetone phosphate, DL‐serine, 1‐stearoyl‐2‐hydroxy‐sn‐glycero‐3‐phosphocholine, Acetylcholine, Choline, Phosphocholine, Triethanolamine
Pyrimidine metabolism	2′‐deoxyuridine 5′‐monophosphate, 3‐ureidopropionic acid, Cytidine, Malonic acid, Methylmalonic acid, Thymine, Uridine, Cytosine, D‐glutamine
Mineral absorption	Alanine, D‐ (+)‐galactose, DL‐serine, DL‐threonine, Glycine, L‐Alanine, L‐methionine, Leucine, Phenylalanine, Phosphoric acid, Tryptophan, D‐glutamine, DL‐asparagine, DL‐proline, DL‐tryptophan, L‐Proline
mTOR signaling pathway	Leucine, Arginine
Oxidative phosphorylation	Fumarate, Phosphoric acid, Succinate
Downregulation in the Glu‐0 group	
Prolactin signaling pathway	DL‐tyrosine, Progesterone, D‐glucose 6‐phosphate, Tyrosine
Glu‐50 vs. Glu‐75	
Upregulation in the Glu‐50 group	
Plant hormone signal transduction	2‐cis‐4‐trans‐abscisic acid, Jasmonic acid, Salicylic acid, Gibberellin a4
cAMP signaling pathway	γ‐aminobutyric acid, Acetylcholine, Prostaglandin i2, Serotonin
D‐Amino acid metabolism	Alanine, Alpha‐ketoglutarate, DL‐Glutamic acid, Glutamic acid, Lysine, Pyruvate, DL‐proline, Histidine
Carbohydrate digestion and absorption	Butanoic acid, D‐ (+)‐galactose, Propanoic acid, Sucrose
Biosynthesis of amino acids	2‐Oxoadipic acid, 3‐dehydroquinic acid, 3‐dehydroshikimate, Alanine, Alpha‐ketoglutarate, Dihydroxyacetone phosphate, DL‐Glutamic acid, DL‐tyrosine, Glutamic acid, L‐Histidinol phosphate, Lysine, N‐α‐acetyl‐l‐ornithine, Pyruvate, Tryptophan, D‐erythro‐imidazolylglycerol phosphate, DL‐proline, DL‐tryptophan, Histidine, L‐citrulline, N2‐Acetyl‐L‐ornithine, Tyrosine
Tyrosine metabolism	DL‐tyrosine, Fumarate, Gentisic acid, P‐coumaric acid, Pyruvate, 3‐methoxytyramine, Hordenine, N‐methyltyramine, Tyramine, Tyrosine
Carbon metabolism	Alanine, Alpha‐ketoglutarate, D‐gluconate, D‐glucono‐1,5‐lactone, Dihydroxyacetone phosphate, DL‐Glutamic acid, Fumarate, Glutamic acid, Pyruvate, β‐d‐glucose
ABC transporters	1,4‐d‐xylobiose, Alanine, D‐galacturonic acid, D‐mannitol, D‐Ribose, DL‐Glutamic acid, Glutamic acid, His‐Lys, Inosine, Lysine, Myo‐inositol, Sucrose, Betaine, Ciprofloxacin, DL‐proline, Histidine
Nicotinate and nicotinamide metabolism	γ‐aminobutyric acid, Dihydroxyacetone phosphate, Fumarate, Nicotinate, Propanoic acid, Pyruvate
Glycine, serine, and threonine metabolism	Pyruvaldehyde, Pyruvate, Tryptophan,1,3‐diaminopropane, Betaine, DL‐tryptophan, Guanidoacetic acid
Pentose phosphate pathway	2‐deoxy‐d‐ribose, D‐gluconate, D‐glucono‐1,5‐lactone, D‐Ribose, Pyruvate,β‐d‐glucose
Mineral absorption	Alanine, D‐(+)‐galactose, Tryptophan, DL‐proline, DL‐tryptophan
Citrate cycle (TCA cycle)	Alpha‐ketoglutarate, Fumarate, Pyruvate
GABAergic synapse	γ‐aminobutyric acid, Alpha‐ketoglutarate, DL‐Glutamic acid, Glutamic acid

Compared to the Glu‐0 group, which had a DA score greater than 0.5 and a *p*‐value less than 0.05, the Glu‐75 group demonstrated alterations in 12 metabolic pathways, with 11 being down‐regulated and 1 up‐regulated. The Prolactin signaling pathway was the sole up‐regulated pathway. In contrast, the down‐regulated pathways included Carbohydrate digestion and absorption, Biosynthesis of amino acids, D‐Amino acid metabolism, Aminoacyl‐tRNA biosynthesis, ABC transporters, Galactose metabolism, Glycerophospholipid metabolism, Pyrimidine metabolism, Mineral absorption, mTOR signaling pathway, and Oxidative phosphorylation. The principal metabolites involved were Alanine, DL‐Glutamic acid, Glutamic acid, DL‐serine, DL‐threonine, Glycine, L‐Alanine, L‐aspartic acid, L‐methionine, Leucine, Lysine, Phenylalanine, Arginine, D‐glutamine, DL‐proline, Histidine, L‐Glutamate, L‐Proline, among others.

Compared to the Glu‐75 group, the Glu‐50 group showed changes in 15 metabolic pathways that were up‐regulated, with a DA score exceeding 0.5 and a *p*‐value below 0.05. The principal up‐regulated pathways included plant hormone signal transduction, cAMP signaling pathway, D‐amino acid metabolism, carbohydrate digestion and absorption, biosynthesis of amino acids, tyrosine metabolism, carbon metabolism, ABC transporters, nicotinate and nicotinamide metabolism, glycine, serine, and threonine metabolism, pentose phosphate pathway, mineral absorption, citrate cycle (TCA cycle), and GABAergic synapse. Consequently, the primary metabolites identified were pyruvate, DL‐glutamic acid, glutamic acid, alpha‐ketoglutarate, γ‐aminobutyric acid, DL‐proline, histidine, D‐ribose, β‐D‐glucose, sucrose, betaine, and propanoic acid, among others.

### 3.6 Effect of MSG Supplementation on Volatile Flavor Compounds in Black Highland Barley

#### 3.6.1 Comparison and Analysis of Volatile Component Profiles in Samples

**FIGURE 4:**
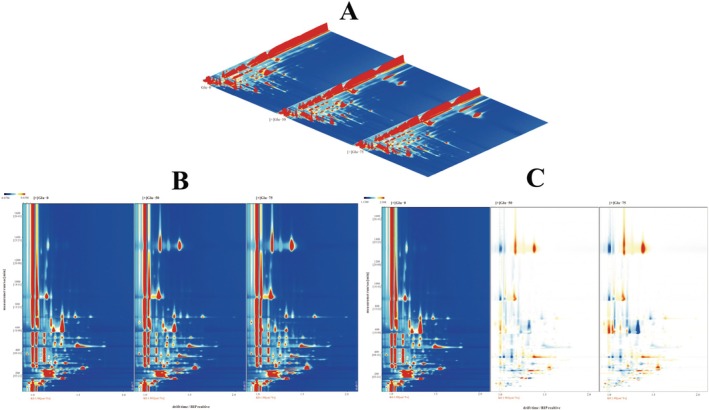
Volatile components GC‐IMS: (A) Three‐dimensional spectrum; (B) Two‐dimensional spectrum; (C) Differential spectrum.

Figure 4A presents the three‐dimensional spectrum of volatile components within the samples, where the axes represent relative migration time (X‐axis), retention time (Y‐axis), and signal peak intensity (Z‐axis). The figure clearly illustrates the variations in volatile organic compounds across different samples. For enhanced clarity, a top‐view perspective is employed for subsequent comparisons. Figure 4B further highlights the distinctions in volatile organic compounds among the different sample groups. To facilitate a more detailed visual comparison of these differences, the spectrum of the Glu‐0 sample was utilized as a reference. Differential comparison charts were generated by subtracting the spectra of the Glu‐50 and Glu‐75 groups from the reference spectrum. In the analysis of volatile organic compounds, a white background following subtraction indicates that the concentration in the target sample is equivalent to that in the reference sample. A red background signifies a higher concentration of the compound in the target sample compared to the reference, whereas a blue background denotes a lower concentration. As illustrated in Figure 4C, relative to the Glu‐0 group, the Glu‐50 and Glu‐75 groups exhibit an increased number of blue spots within the drift time interval of 1.0–1.3 ms, alongside a limited number of red spots within the intervals of 1.0–1.3 ms and 1.0–1.6 ms.

#### 3.6.2 Qualitative and Quantitative Analysis of Volatile Flavor Compounds in Black Highland Barley With Exogenous MSG Addition

As illustrated in Table 6, the analysis identified a total of 57 volatile compounds in black highland barley following the addition of exogenous MSG. Of these, 12 volatile compounds lacked corresponding matching information and thus could not be definitively identified at this time. The remaining 45 compounds were categorized as follows: 18 alcohols, 7 ketones, 7 esters, 6 carboxylic acids, 4 heterocyclic compounds, 2 aldehydes, and 1 thioether. Alcohols, ketones, and esters were the most prevalent, followed by carboxylic acids, while aldehydes and other compounds were the least abundant. Compared to the Glu‐0 control group, adding external MSG significantly raised the levels of aromatic flavor compounds, such as ethyl butyrate, 2‐butanol, and 1‐hexanol D (*p* < 0.05), while significantly decreasing the concentration of pungent odor compounds, including 1‐hydroxy‐2‐propanone and methyl acetate.

**TABLE 6:** Effect of exogenous MSG addition on the composition and content of volatile compounds in black highland barley.

Item	Compound	Relative content (%)		
		Glu‐0	Glu‐50	Glu‐75
Alcohols	(Z)‐2‐Penten‐1‐ol	0.29 ± 0.01a	0.42 ± 0.03b	0.53 ± 0.02a
	1,2‐Ethanediol	0.26 ± 0.02a	0.23 ± 0.02ab	0.21 ± 0.01b
	1‐Hexanol dimer	0.65 ± 0.03c	0.90 ± 0.04b	1.14 ± 0.11a
	1‐Hexanol monomer	2.04 ± 0.02a	1.78 ± 0.03ab	1.71 ± 0.11b
	1‐Hexanol polymer	0.05 ± 0.01b	0.07 ± 0.01b	0.10 ± 0.02a
	1‐Pentanol dimer	0.79 ± 0.03a	0.75 ± 0.03a	0.63 ± 0.01b
	1‐Pentanol monomer	0.85 ± 0.03a	0.85 ± 0.01a	0.68 ± 0.02b
	1‐Penten‐3‐ol	0.48 ± 0.02a	0.52 ± 0.00a	0.42 ± 0.04b
	2‐Butanol	1.08 ± 0.03c	2.80 ± 0.09a	2.30 ± 0.03b
	2‐Methyl‐1‐propanol dimer	1.65 ± 0.06a	0.79 ± 0.03b	0.72 ± 0.04b
	2‐Methyl‐1‐propanol monomer	0.60 ± 0.06a	0.50 ± 0.02b	0.43 ± 0.03b
	2‐Pentanol	0.17 ± 0.01b	0.18 ± 0.01b	0.28 ± 0.01a
	3‐Methyl‐1‐pentanol	0.10 ± 0.02a	0.03 ± 0.01b	0.03 ± 0.01b
	3‐Methylbutan‐1‐ol dimer	6.25 ± 0.14a	3.87 ± 0.10b	3.99 ± 0.13b
	3‐Methylbutan‐1‐ol polymer	0.45 ± 0.01a	0.26 ± 0.01c	0.30 ± 0.03b
	Butanol dimer	0.40 ± 0.01c	2.49 ± 0.07a	2.11 ± 0.01b
	Butanol polymer	0.01 ± 0.01c	0.18 ± 0.01a	0.13 ± 0.01b
	Ethanol	16.06 ± 0.62a	12.63 ± 0.30b	11.75 ± 0.15c
Ketone	1‐Hydroxy‐2‐propanone	3.17 ± 0.19a	1.58 ± 0.12b	0.89 ± 0.12c
	2‐Butanone	0.62 ± 0.04b	1.76 ± 0.15a	0.66 ± 0.03b
	3‐Hexanone	0.12 ± 0.04a	0.07 ± 0.01b	0.06 ± 0.00b
	3‐Hydroxy‐2‐butanone	18.65 ± 0.27a	11.52 ± 0.81b	3.39 ± 1.56c
	3‐Nonanone	0.14 ± 0.02a	0.12 ± 0.01a	0.06 ± 0.01b
	3‐Pentanone	1.00 ± 0.13a	0.67 ± 0.02b	0.62 ± 0.03b
	Acetone	5.89 ± 0.14a	5.03 ± 0.26b	4.12 ± 0.08c
Ester	3‐Methylbutyl butanoate	0.02 ± 0.00b	0.02 ± 0.01b	0.04 ± 0.01a
	Acetic acid ethyl ester	7.00 ± 0.05a	4.10 ± 0.11c	5.34 ± 0.20b
	Acetic acid propyl ester	0.07 ± 0.00c	0.14 ± 0.01a	0.12 ± 0.01b
	Butanoic acid ethyl ester	6.37 ± 0.37c	7.62 ± 0.25b	8.58 ± 0.05a
	Ethyl caproate	0.10 ± 0.01a	0.07 ± 0.01b	0.09 ± 0.01ab
	Methyl acetate	0.18 ± 0.02a	0.08 ± 0.01b	0.14 ± 0.05ab
	Methyl pentanoate	0.12 ± 0.01c	0.24 ± 0.02b	0.45 ± 0.05a
Carboxylic acids	1‐Butanoic acid dimer	0.69 ± 0.13c	5.53 ± 1.04b	12.01 ± 0.51a
	1‐Butanoic acid monomer	3.02 ± 0.39c	9.24 ± 0.61b	11.64 ± 0.23a
	2‐Methyl propanoic acid	0.48 ± 0.06a	0.35 ± 0.10ab	0.23 ± 0.03b
	Acetic acid dimer	5.27 ± 0.88c	8.49 ± 0.68b	10.17 ± 0.20a
	Acetic acid monomer	10.60 ± 0.29a	9.65 ± 0.10b	9.65 ± 0.07b
	Propanoic acid	0.05 ± 0.01b	0.06 ± 0.01b	0.09 ± 0.01a
Heterocyclic	2,4,5‐Trimethylthiazole	0.20 ± 0.04a	0.08 ± 0.02b	0.10 ± 0.01b
	2,5‐Dimethylpyrazine	0.32 ± 0.01a	0.16 ± 0.01b	0.07 ± 0.02c
	2‐Pentylfuran	0.97 ± 0.12a	0.87 ± 0.07b	0.69 ± 0.06b
	Pyrrolidine	0.14 ± 0.02a	0.07 ± 0.01b	0.08 ± 0.00b
Aldehyde	Butanal	1.07 ± 0.05c	1.58 ± 0.02a	1.29 ± 0.02b
	Propanal	0.50 ± 0.03b	0.49 ± 0.05b	0.71 ± 0.06a
Thiol ether	Dimethyl sulfide	1.06 ± 0.02b	1.12 ± 0.03ab	1.26 ± 0.11a

*Note:* Values are presented as mean ± SD. Different lowercase superscript letters within the same row indicate significant differences among treatments (*p* < 0.05).

**FIGURE 5:**
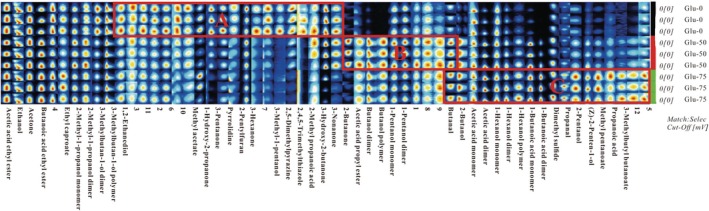
Fingerprint of volatile components.

A detailed comparative analysis of volatile compounds in the three different samples was undertaken, accompanied by a comprehensive fingerprint analysis of all volatile constituents, as depicted in Figure 5. The results of this comparative analysis, in conjunction with the subsequent Principal Component Analysis (PCA) plot, revealed the identification of 57 peaks across the three samples. Of these, 37 substances were qualitatively characterized, encompassing a range of chemical classes such as aldehydes, alcohols, esters, and ketones. Notably, the volatile compounds in the Glu‐0 sample exhibited the most significant variation. In the figure, compounds with elevated concentrations in specific samples are clustered together. For instance, the compounds located in area A of the figure were found in higher concentrations in the Glu‐0 sample, predominantly consisting of methyl‐1‐propanol, 3‐methylbutanol, ethylene glycol, methyl acetate, 1‐hydroxy‐2‐propanone, 3‐pentanone, and pyridine. Conversely, the compounds in area B exhibited the highest concentrations in the Glu‐50 sample, primarily including propyl acetate, butanol, pentanol, and butyraldehyde. Meanwhile, the compounds in area C were most concentrated in the Glu‐75 sample, mainly comprising 2‐butanol, acetic acid, and hexanol.

**FIGURE 6:**
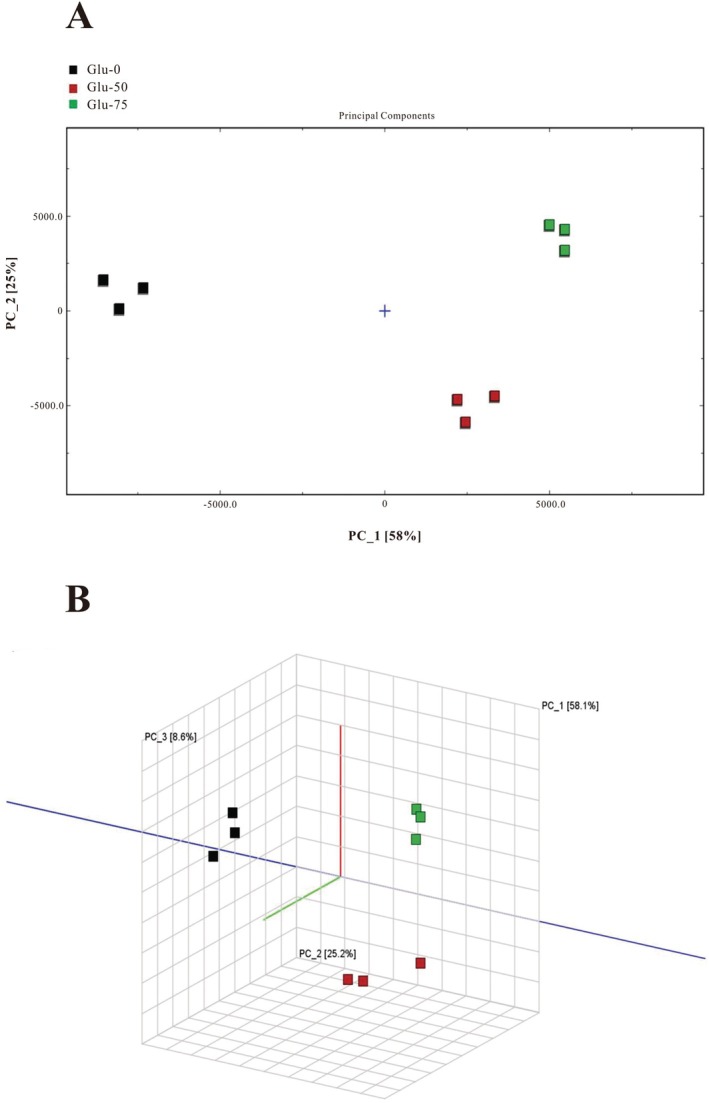
PCA score plot of volatile components.

Principal Component Analysis (PCA) was conducted on the gas chromatography‐ion mobility spectrometry data utilizing SIMCA 14.1 software. The resultant PCA plot (refer to Figure 6) illustrates the distinctions among the various samples: a shorter distance between points indicates a smaller difference, whereas a greater distance signifies a more pronounced difference. Statistical analysis of the volatile components across the three samples revealed that PCA effectively discriminated between the samples. The principal components had the following contribution rates: PC1 accounted for 58.1%, PC2 for 25.2%, and PC3 for 8.6%. These findings suggest that these three principal components adequately represent the volatile flavor substances present in the samples.

#### 3.6.3 Identification of Key Flavor Substances in Black Highland Barley With Exogenous Addition of MSG

To elucidate the contribution of volatile compounds from black highland barley, with the exogenous addition of MSG, to the overall flavor profile of black highland barley, Table 7 presents the ROAV values of volatile substances in black highland barley at varying concentrations of MSG. A higher ROAV value signifies a more substantial contribution of the component to the sample's overall flavor. Components with ROAV values exceeding 1 are identified as key flavor substances, whereas those with ROAV values ranging from 0.1 to less than 1 are considered modifiable flavor substances.

As illustrated in Table 7, a total of 14 distinctive flavor compounds were identified in black highland barley. Notably, 1‐Butanoic acid monomer was found to have a relatively high concentration, possessing a sensory threshold of 0.001 μg/kg and characterized by strong acetic acid, cheese, butter, and fruity aroma notes. This compound significantly contributes to the overall flavor profile of black highland barley, warranting its ROAV to be set at 100. Additionally, 1‐Butanoic acid dimer, 3‐Hydroxy‐2‐butanone, Dimethyl sulfide, and Butanal are identified as the primary flavor compounds (ROAV ≥ 1) that constitute the aroma of highland barley and are the principal factors responsible for intergroup variations. Furthermore, the Relative Odor Activity Value (ROAV) of the 1‐Butanoic acid dimer exhibits an upward trend with increasing concentrations of Glutamate (Glu). In contrast, the ROAV values for compounds such as 2,4,5‐Trimethylthiazole, 2‐Methyl propanoic acid, 3‐Nonanone, 3‐Methylbutan‐1‐ol dimer, Propanal, Butanoic acid ethyl ester, Ethyl caproate, Methyl pentanoate, and Pyrrolidine are higher in the control group (Glu‐0) compared to the Glu‐50 and Glu‐75 groups, indicating their role in modulating the flavor profile of these latter groups within the range of 0.1 ≤ ROAV ≤ 1. Additionally, the ROAV of several compounds, including 3‐Hydroxy‐2‐butanone and Dimethyl sulfide, tends to decrease as the Glu concentration increases.

**TABLE 7:** Key volatile flavor compounds in black highland barley under different concentrations of exogenous MSG addition.

Item	CAS	Threshold μg/kg	ROAV			Odor characteristics
			Glu‐0	Glu‐50	Glu‐75	
1‐Butanoic acid monomer	107–92‐6	0.001	100.00	100.00	100.00	Cheese, butter, fruity
1‐Butanoic acid dimer	107–92‐6	0.001	22.83	59.89	103.18	Cheese, butter, fruity
3‐Hydroxy‐2‐butanone	513–86‐0	0.055	11.18	2.26	0.53	Butter, cream
Dimethyl sulfide	75–18‐3	0.009	3.74	1.29	1.15	Cabbage, sulfur, gasoline
Butanal	123–72‐8	0.013	2.73	1.32	0.85	Pungent, fruity, green leaf
2,4,5‐Trimethylthiazole	13,623–11‐5	0.002	3.61	0.50	0.46	Cocoa, chocolate, caramel, nutty
2‐Methyl propanoic acid	79–31‐2	0.005	3.19	0.76	0.40	Yogurt, rancid cream
3‐Nonanone	925–78‐0	0.002	2.70	0.78	0.33	Fresh, sweet, jasmine, spicy, herb, fruity
3‐Methylbutan‐1‐ol dimer	123–51‐3	0.100	2.06	0.42	0.34	Whiskey, banana, fruity
Propanal	123–38‐6	0.020	0.83	0.26	0.30	Pungent, green grassy
Butanoic acid ethyl ester	105–54‐4	0.280	0.75	0.29	0.26	Pineapple, fruity, ester, whiskey
Ethyl caproate	123–66‐0	0.004	0.72	0.16	0.17	Pineapple, fruity, wine
Methyl pentanoate	624–24‐8	0.011	0.36	0.23	0.35	Ammoniacal
Pyrrolidine	123–75‐1	0.015	0.32	0.05	0.05	Fruity

### 3.7 Integrated Correlation Analysis

In the investigation of nontarget metabolites in black highland barley subjected to varying concentrations of MSG, pairwise comparative analyses among sample groups are essential. Differential metabolites between the two groups are mainly identified by a VIP score greater than 1. A higher VIP score signifies a greater contribution of the metabolite to the differentiation between groups. To improve the accuracy of screening and decrease false positives, applying the criteria of a Fold Change (FC) over 1.5 along with a *p*‐value under 0.05 is suggested. This approach ensures that the identified differential metabolites possess both statistical significance and biological relevance.

**FIGURE 7:**
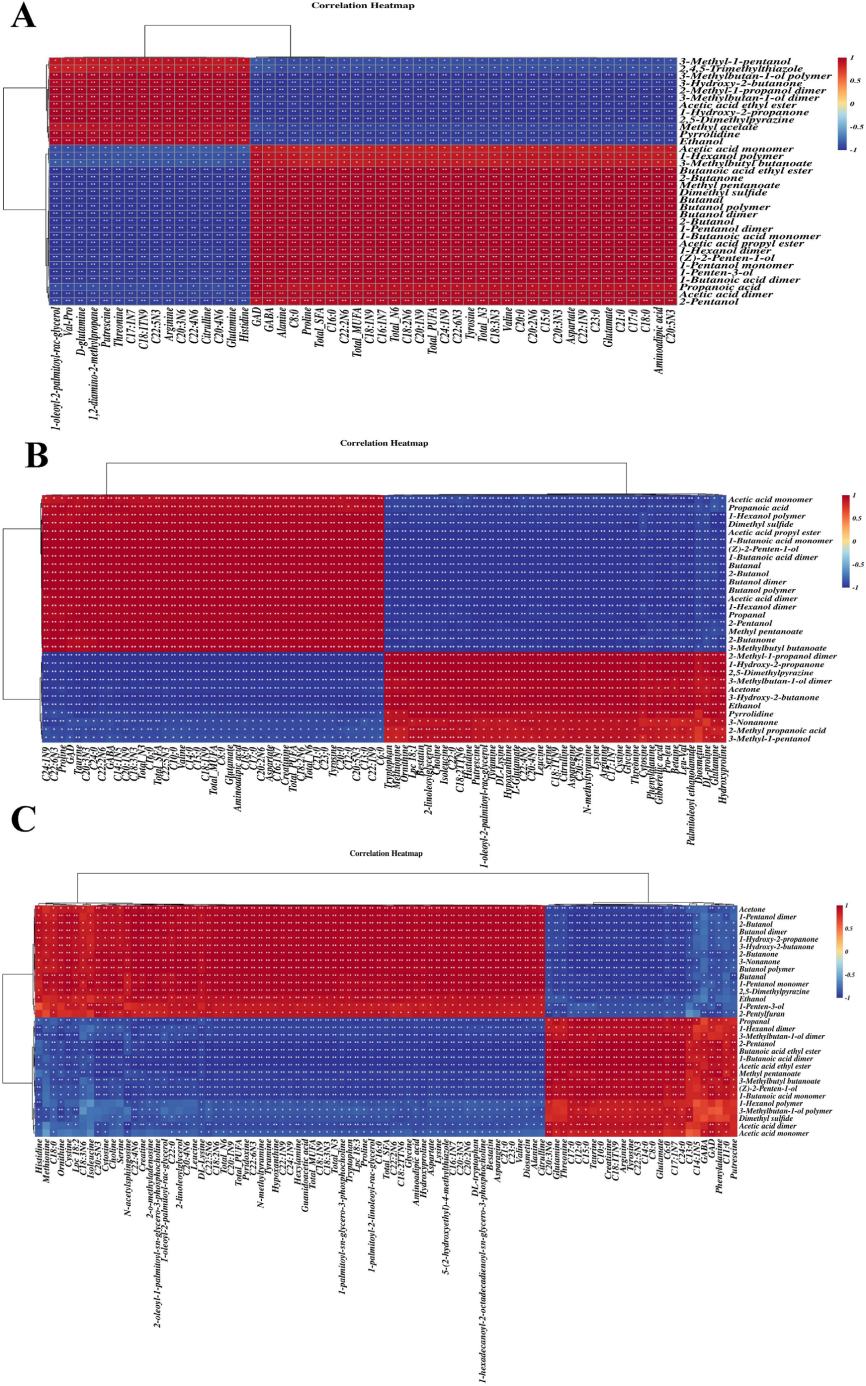
Correlation analysis heatmaps among amino acids, fatty acids, untargeted metabolites, and volatile compounds in black highland barley under different MSG treatments: (A) Glu‐0 vs. Glu‐50; (B) Glu‐0 vs. Glu‐75; (C) Glu‐50 vs. Glu‐75.

As illustrated in Figure 7, the heatmap effectively visualizes the results of the correlation analysis among amino acids, fatty acids, nontarget metabolites, and volatile flavor compounds in black highland barley subjected to varying concentrations of MSG treatments. In the correlation heatmap depicted in Figure 7, which compares Glu‐0 vs. Glu‐50, Glu‐0 vs. Glu‐75, and Glu‐50 vs. Glu‐75, red indicates a positive correlation, with darker shades signifying a stronger positive correlation. This suggests that the content variation trends of the two substances are more consistent. Conversely, blue denotes a negative correlation, with stronger negative correlations indicating more divergent content variation trends between the two substances.

Figure 7A presents a correlation analysis heatmap comparing the conditions of 0 mmol・L^−1^ and 50 mmol・L^−1^ MSG in black highland barley. The study shows a strong correlation between amino acids, fatty acids, nontarget metabolites, and volatile flavor compounds in these conditions. Specifically, there is a strong positive correlation between the following substances: GAD, GABA, Alanine, C8:0, Proline, Total SFA, C16:0, C22:2 N6, Total MUFA, C18:1 N9, C16:1 N7, Total N6, C18:2 N6, C18:1 N9, Total PUFA, C24:1 N9, C22:6 N3, Tyrosine, Total N3, C18:3 N3, Valine, C20:0, C20:2 N6, C15:0, C20:3 N3, Aspartate, C22:1 N9, C23:0, Glutamate, C21:0, C17:0, C18:0, Aminoadipic acid, and C20:5 N3. Additionally, a strong positive correlation is observed with Acetic acid monomer, 3‐Methyl‐1‐pentanol, Butanoic acid butanoate, 2‐Butanone ethyl ester, Methyl pentanoate, Dimethyl sulfide, Butanal, Butanol polymer, Butanol dimer, 2‐Butanol, 1‐Butanol dimer, 1‐Butanoic acid monomer, Acetic acid propyl ester, (Z)‐2‐Penten‐1‐ol, 1‐Hexanol dimer, 1‐Pentanol monomer, 1‐Penten‐3‐ol, 1‐Butanoic acid dimer, Propanoic acid dimer, Acetic acid dimer, and 2‐Pentanol.

Figure 7B presents a heatmap of the correlation analysis between Glu‐0 and Glu‐75. The analysis reveals a highly significant correlation among GABA, GAD, total N3, total N6, total SFA, total MUFA, glutamate, proline, and various volatile compounds, including 2‐Methyl‐1‐propanol dimer, 1‐Hydroxy‐2‐propanone, 3,5‐Dimethylpyrazine, 2‐Methylbutan‐1‐ol dimer, acetone, 3‐Hydroxy‐2‐butanone, ethanol, pyrrolidine, 2‐Nonanone, 3‐Methyl propanoic acid, and 3‐Methyl‐1‐pentanol. At the same time, these volatile flavor compounds show a strong negative correlation with most amino acid compounds.

Figure 7C presents a correlation analysis heatmap illustrating the relationship between Glu‐50 and Glu‐75. The analysis reveals a strong correlation between the targeted metabolites and the volatile flavor compounds derived from amino acids and fatty acids in black highland barley at concentrations of 50 mmol・L^−1^ and 75 mmol・L^−1^. Notably, there is an extremely significant correlation between the following compounds: C20:3 N6, glutamine, threonine, C17:0, C12:0, taurine, C15:0, C10:0, creatinine, C18:1 N9, arginine, tyrosine, C22:5 N3, C14:0, glutamate, C8:0, C24:0, C17:1 N7, C23:0, C14:1 N5, GABA, GAD, phenylalanine, C11:0, putrescine, and the volatile compounds propanal, 1‐hexanol dimer, 3‐methylbutan‐1‐ol dimer, 2‐pentanol, butanoic acid ethyl ester, 1‐butanoic acid dimer, acetic acid ethyl ester, methyl pentanoate, 3‐methylbutyl butanoate, (Z)‐2‐penten‐1‐ol, 1‐butanoic acid monomer, 1‐hexanol polymer, 3‐methylbutan‐1‐ol polymer, dimethyl sulfide, acetic acid dimer, and acetic acid monomer.

## 4 Discussion

### 4.1 Germination as a Strategy for GABA Enrichment and the Rationale for MSG Selection

Germination is a well‐documented bioprocess that improves the nutritional and functional properties of cereal grains by reactivating dormant metabolic pathways. This process facilitates the mobilization of storage reserves and stimulates the synthesis of bioactive compounds, such as gamma‐aminobutyric acid (GABA), phenolic compounds, and free amino acids, while concurrently reducing anti‐nutritional factors. (Karki et al. 2025; Sharma et al. 2017). In contrast to chemical synthesis or microbial fermentation, germination is regarded as an environmentally sustainable, safe, and cost‐effective method for the natural enrichment of GABA in plant‐based matrices (Sarasa et al. 2020). Specifically, within the context of black highland barley (BHB), germination provides a viable framework for exploring precursor‐driven GABA accumulation and its related metabolic effects.

In the cytoplasm, GABA is produced through two main routes: the GABA shunt and the polyamine breakdown pathway (Hu et al. 2024). A pivotal factor affecting GABA synthesis during germination is the availability of its direct precursor, L‐glutamate. Given the limited availability of endogenous glutamate, exogenous supplementation can significantly enhance the substrate supply for glutamate decarboxylase (GAD). Among various exogenous substances, Jiang et al. (2021) identified that a concentration of 2.0 mg·mL^−1^ of MSG was optimal for application, resulting in a 1.22‐fold increase in GABA content compared to the control group. Li et al. (2019) further demonstrated that utilizing MSG as a substrate for GABA production not only enhances GABA yield compared to free glutamate but also reduces production costs, suggesting that MSG serves as an effective exogenous additive. It is hypothesized that the efficacy of MSG as an exogenous additive is primarily due to its significantly higher water solubility and dissociation efficiency compared to free glutamic acid at room temperature, which facilitates rapid uptake by germinating grains (Schmidt et al. 2020). Second, MSG is a widely accessible, food‐grade additive, which enhances the translational relevance for potential industrial applications (Yamamoto and Inui‐Yamamoto 2023). Third, in comparison to glutamine or other glutamate derivatives, MSG provides a pure and direct source of glutamate, devoid of additional nitrogenous side chains that could complicate metabolic interpretations. MSG, the sodium salt of glutamic acid, was chosen as the exogenous precursor in this study due to several physicochemical and practical considerations.

In this investigation, BHB grains were germinated under controlled conditions with varying concentrations of MSG supplementation (0–75 mmol·L^−1^). Based on preliminary dose–response experiments, two concentrations (50 and 75 mmol·L^−1^) that resulted in significant GABA enrichment were selected for comprehensive targeted and untargeted metabolomic analyses. The subsequent sections explore the impact of MSG treatment on GABA accumulation, amino acid and fatty acid profiles, and volatile flavor compounds, with a focus on correlational patterns and hypothetical mechanisms within the plant metabolic framework.

### 4.2 MSG Effects on GABA Accumulation and Amino Acid Metabolism

Germination enhances GABA content by activating the key enzyme glutamate decarboxylase (GAD); however, the regulation of external stimuli can further augment this accumulation (Minchán‐Velayarce et al. 2025). Among these stimuli, the regulation of minerals and trace elements during germination can significantly increase GABA levels. For instance, Sun et al. (2024), demonstrated that soaking millet seeds in a 60 mg/L Na2SeO3 solution and spraying them with a 2 mg/L Na2SiO3 solution significantly enriched GABA content. Additionally, the application of exogenous sodium chloride has been shown to enhance GABA levels in plants (Oh et al. 2019). As illustrated in Table 2, varying concentrations of exogenous MSG significantly influenced GABA and GAD levels in black highland barley. Specifically, the Glu‐75 group exhibited the highest GABA content (2.55 mg/g) and GAD activity (25.476 U/g·h). In the absence of exogenous MSG, GABA synthesis relies entirely on endogenous glutamate. Notably, the positive correlation between GABA and glutamate levels observed in this study aligns with previous findings (Rideaux et al. 2022). Jin et al. (2022) demonstrated that MSG functions as an effective substrate for GABA synthesis in mulberry leaves under stress conditions. Conversely, Y. Sun et al. (2022) indicated that although GABA production is achievable in laboratory‐scale culture media containing MSG, its application within the food industry has not yet been realized.

Given that GAD utilizes glutamate as its direct substrate, the exogenous administration of MSG—once absorbed and converted to glutamate—has the potential to affect intracellular amino acid metabolism on a broader scale (Shen et al. 2025). As illustrated in Table 3, the introduction of exogenous MSG significantly altered both the composition and concentration of amino acids. Specifically, the glutamate concentration in the Glu‐75 group reached 299,976 μmol/L, which was substantially higher than that observed in the other two groups, whereas glutamine levels exhibited a declining trend. From the perspective of plant metabolism, glutamine functions as a nitrogen donor and a precursor for glutamate through the activity of glutaminase, and its depletion may indicate an increased demand for glutamate in the synthesis of GABA (Feehily and Karatzas 2013; Peng et al. 1994). The Glu‐75 group demonstrated a significantly elevated total amino acid content as well as umami amino acid content in comparison to the other groups. In alignment with these results, Jiang et al. (2023) reported that the total amino acid content in red beans treated with MSG increased by a factor of 4.44, with significant increases observed in 21 amino acids, excluding homocysteine. Similarly, Wu et al. (2020) found that the exogenous application of sodium and other compounds enhanced GAD activity and amino acid content in tomato leaves. Collectively, these findings imply that exogenous MSG may affect pathways related to amino acid decomposition and biosynthesis, although direct evidence of changes in metabolic flux has yet to be established.

### 4.3 Influence of MSG on Fatty Acid Profiles and Flavor Development

Amino acids are crucial contributors to the umami taste in food, while lipids serve as essential precursors for a variety of volatile flavor compounds, including aldehydes, ketones, and lactones. Following the confirmation that Glu‐75 treatment significantly enhanced umami‐related amino acids, we proceeded to investigate changes in fatty acid profiles. As illustrated in Table 4, the administration of exogenous MSG had a pronounced impact on both the content and diversity of fatty acids. In particular, the Glu‐50 group had significantly higher levels of saturated fatty acids (SFA), monounsaturated fatty acids (MUFA), polyunsaturated fatty acids (PUFA), and n‐3/n‐6 fatty acids compared to the other groups. Previous research has indicated that sodium chloride concentrations can affect fatty acid composition; higher sodium levels may inhibit the synthesis of certain fatty acids, while lower sodium levels may enhance diversity (Salaberría et al. 2021). Furthermore, Jiang et al. (2023) observed that exogenous intervention with MSG resulted in an increase in the concentrations of linoleic acid and linolenic acid in red beans by 11.63% and 25.46%, respectively, compared to their levels prior to germination. The scientists suggested that oleic acid might be transformed into linoleic acid by the enzyme 12‐desaturase, and then linoleic acid could be further processed into docosahexaenoic acid (DHA) through multiple metabolic reactions. Additionally, Jiang et al. (2021) found that treatment with vacuum and MSG can induce the expression of certain GAD genes (VaGAD and VaPAO), with these gene expressions being positively correlated with GABA content and enzyme activity. Consequently, we hypothesize that the alterations in unsaturated fatty acids observed in the Glu‐50 cohort may be attributable to the exogenous addition of MSG. However, further investigation is required to elucidate the precise correlation and underlying mechanisms.

Volatile compounds in barley primarily arise from the oxidation of lipids and the degradation of proteins, carbohydrates, and amino acids, leading to the production of alcohols, esters, aldehydes, ketones, and alkanes (Wang et al. 2025a). Previous research has indicated that grain germination can enhance the flavor profiles of baked products (Liu et al. 2022; Ma et al. 2025). Additionally, Du et al. (2022) reported significant increases in gamma‐aminobutyric acid (GABA), total phenols, total flavonoids, essential amino acids, and aroma compounds in germinated highland barley. In our study, compared to the control group (without MSG), aroma compounds such as 1‐hexanol, butanol, acetic acid propyl ester, butanoic acid ethyl ester, methyl pentanoate, 1‐butanoic acid, acetic acid dimer, and butanal were elevated following MSG supplementation. This enhancement may be linked to the degradation of umami amino acids and fatty acids. In contrast to the findings of Almaguer et al. (2024), who reported undesirable odors during quinoa germination, our study did not detect an increase in such off‐flavor compounds in the Glu‐50 and Glu‐75 groups.

A Pearson correlation analysis was conducted to investigate the relationships among differential amino acids, fatty acids, gamma‐aminobutyric acid (GABA), glutamate decarboxylase (GAD), and volatile compounds across three distinct groups. The observed elevations in GABA levels may indicate increased activity of the GABA shunt, potentially correlating with the synthesis of amino acids and fatty acids. As illustrated in Figure 7A, both polyunsaturated fatty acids (PUFA) and monounsaturated fatty acids (MUFA) demonstrated positive correlations with butanol and butanal. In the Glu‐50 and Glu‐75 groups, glutamate showed significant positive correlations with 1‐hexanol, acetic acid propyl ester, methyl pentanoate, 1‐butanoic acid, and acetic acid dimer. In contrast, fatty acids such as C17:1 N7, C20:3 N6, C18:1TN9, and C22:5 N3 exhibited significant positive correlations with these volatile compounds in the Glu‐75 group, while displaying significant negative correlations in the Glu‐0 group. Wang et al. (2025b) identified a significant correlation between fatty acids and flavor during the thermal processing of black highland barley. It is posited that the fatty acids present in black highland barley undergo oxidative degradation during heat treatment, resulting in the formation of hexenal and (E, E)‐2,4‐dodecadienal aldehyde. Research has shown that pretreatment processes effectively inactivate lipase, lipoxygenase, and peroxidase enzymes in wheat, thereby reducing the formation of lipid oxidation markers, including flavor compounds such as 3‐methylbutane and hexanoic acid (Zhang et al. 2023). These correlational patterns suggest that changes in flavor compounds following the addition of MSG are linked to alterations in amino acids and fatty acids. However, the formation of volatile compounds may also result from multiple concurrent processes, including lipid oxidation during germination stress, which may occur independently of MSG treatment.

### 4.4 Untargeted Metabolomic Insights Into MSG Treatment

Building upon the targeted metabolomic analysis of amino acids and fatty acids, we conducted an untargeted metabolomic investigation on black highland barley exposed to varying concentrations of MSG. As detailed in Table S1, the comparison between Glu‐0 and Glu‐50 conditions revealed 238 differential metabolites, with approximately 30% identified as amino acids and fatty acids. The analysis of Glu‐0 versus Glu‐75 conditions identified 269 differential metabolites, of which around 31% were amino acids and fatty acids. Similarly, the Glu‐50 versus Glu‐75 comparison identified 238 differential metabolites, with approximately 33% comprising amino acid and fatty acid compounds. These findings suggest that amino acid and lipid metabolism are among the primary metabolic networks responding to MSG supplementation, aligning with the observed alterations in targeted metabolites. This is consistent with previous research by Kumar et al. (2019), which demonstrated that exogenous gamma‐aminobutyric acid (GABA) can modulate the metabolic pathways of fatty acids and amino acids.

A KEGG pathway enrichment analysis was performed on the differential metabolites to explore the affected metabolic pathways further. As illustrated in Figure 3, the Glu‐50 group, in comparison to the Glu‐0 group, exhibited significant enrichment of differential metabolites in pathways such as butanoate metabolism, histidine metabolism, the citrate cycle (TCA cycle), and pyruvate metabolism. Of particular note, the butanoate metabolism pathway, where GABA serves as a key intermediate, was among the most significantly affected. There was a marked increase in amino acid‐related metabolites and GABA itself within the Glu‐50 group. In contrast, the Glu‐75 group, relative to the Glu‐0 group, demonstrated a downregulation of amino acid biosynthesis pathways. A direct comparison between the Glu‐75 and Glu‐50 groups revealed that pathways including amino acid biosynthesis, glycine‐serine–threonine metabolism, the TCA cycle, and the GABA shunt were comparatively downregulated in the Glu‐75 group. Although both the Glu‐50 and Glu‐75 groups exhibited downregulation in amino acid synthesis and metabolism pathways compared to the Glu‐0 group, the exogenously added MSG dissociated into glutamate under specific pH conditions, resulting in a glutamate concentration that was several times higher than that of other amino acids. Furthermore, glutamate decarboxylase (GAD) utilizes glutamate as a substrate and facilitates its decarboxylation to produce gamma‐aminobutyric acid (GABA), with the coenzyme pyridoxal 5′‐phosphate (PLP) playing an essential role in this process (Huang et al. 2018). This study observed that the addition of 50% and 75% MSG led to a three‐ to four‐fold increase in GABA content. It is hypothesized that the introduction of MSG enhances both the activity and gene expression of GAD, as well as the gene expression of calmodulin. This enhancement likely promotes the conversion of glutamate to GABA and improves the efficiency of the GABA shunt in increasing GABA levels.

### 4.5 Limitations and Future Directions

There are several limitations in this study that need to be recognized. Firstly, although we propose that MSG enhances GAD activity, gene expression, calmodulin signaling, and GABA shunt flux, we did not directly assess GAD gene expression through quantitative polymerase chain reaction (qPCR), intracellular calcium ion (Ca^2+^) concentrations, or metabolic flux via isotopic tracer methodologies. Consequently, our mechanistic assertions remain speculative and necessitate validation through transcriptomic analysis, calcium imaging, and flux analysis techniques. Secondly, the lack of a sodium chloride control (e.g., equimolar Na^+^) precludes the definitive attribution of the observed MSG effects to glutamate alone, as sodium‐induced osmotic or ionic stress may also contribute to the metabolic alterations observed. We therefore recommend that future investigations incorporate appropriate sodium controls to disentangle MSG‐specific effects from those attributable to sodium. Thirdly, our Pearson correlation analyses do not infer metabolic directionality. The associations presented herein should be regarded as exploratory and hypothesis‐generating rather than confirmatory. In conclusion, this study elucidates the complex interplay among processing parameters, MSG supplementation, metabolic transformations, flavor quality, and health attributes, while acknowledging the limitations previously discussed. Future research should consider incorporating metatranscriptomics and metabolic flux analysis to investigate the impact of MSG on metabolic pathways and microbial communities during the germination of black highland barley. This approach could facilitate the targeted optimization of both flavor and nutritional quality.

## 5 Conclusion

In black highland barley, the supplementation of exogenous MSG proved more effective in promoting GABA accumulation compared to direct supplementation with glutamic acid. Analysis of the metabolite profiles and established biochemical pathways suggests that MSG may dissociate into glutamic acid, which can then act as a substrate for the enzyme glutamate decarboxylase in the presence of pyridoxal 5′‐phosphate as a coenzyme, facilitating GABA synthesis. The metabolomic alterations identified in this study align with the involvement of the GABA shunt and indicate potential connections with tricarboxylic acid cycle‐related metabolism. These connections may contribute to changes in unsaturated fatty acids, amino acid derivatives, and metabolites associated with flavor during the germination process. Volatile profiling further suggested that MSG treatment was correlated with an increased abundance of several key aroma‐related compounds, without the detectable accumulation of commonly reported off‐flavor compounds under the analytical conditions employed. However, these mechanistic associations were inferred from metabolite patterns and existing biochemical knowledge rather than being directly demonstrated through gene expression analysis, metabolic flux analysis, isotopic tracing, or direct measurements of TCA cycle activity. Overall, these findings provide evidence that MSG‐assisted germination is a promising strategy for enhancing GABA accumulation and modulating quality‐related metabolites in black highland barley, indicating its potential utility in the development of GABA‐enriched cereal ingredients and precursor‐assisted processing approaches.

## Author Contributions

**Nana Ma:** validation, formal analysis, investigation, resources, data curation, writing – original draft, writing – review and editing, supervision. **Xiaohang Lu:** methodology, conceptualization, investigation, resources, writing – original draft, writing – review and editing, supervision, project administration, funding acquisition. **Yuan Wang:** software, formal analysis, data curation, writing – original draft, visualization.

## Funding

This research was funded by Independent research projects of the State Key Laboratory of Qinghai University (2024‐ZZ‐06).

## Ethics Statement

The authors have nothing to report.

## Conflicts of Interest

The authors declare no conflicts of interest.

## Supporting information


**Figure S1:** Overlaid total ion chromatograms of QC samples in positive (A) and negative (B) ion modes.
**Table S1:** Differential metabolites identified in Glu‐0, Glu‐50, and Glu‐75 groups.

## Data Availability

The data that support the findings of this study are available on request from the corresponding author.

## References

[fsn372125-bib-0001] Ahmad, S. , and Q. Fariduddin . 2024. “Deciphering the Enigmatic Role of Gamma‐Aminobutyric Acid (GABA) in Plants: Synthesis, Transport, Regulation, Signaling, and Biological Roles in Interaction With Growth Regulators and Abiotic Stresses.” Plant Physiology and Biochemistry 208: 108502. 10.1016/j.plaphy.2024.108502. 38492486

[fsn372125-bib-0002] Almaguer, C. , H. Kollmannsberger , M. Gastl , and T. Becker . 2024. “Influence of the Malting Conditions on the Modification and Variation in the Physicochemical Properties and Volatile Composition of Barley ( *Hordeum vulgare* L.), Rye ( *Secale cereale* L.), and Quinoa ( *Chenopodium quinoa* Willd.) Malts.” Food Research International 196: 114965. 10.1016/j.foodres.2024.114965. 39614532

[fsn372125-bib-0003] Bai, T. , Y. Jin , M. Zhu , and B. Wang . 2019. “Effect of Altitude Difference on Quality of Highland Barley Varieties.” Journal of the Chinese Cereals and Oils Association 34, no. 2: 34–39. https://kns.cnki.net/KCMS/detail/detail.aspx?dbcode=CJFQ&dbname=CJFDLAST2019&filename=ZLYX201902008.

[fsn372125-bib-0004] Benidickson, K. H. , L. M. Raytek , G. J. Hoover , et al. 2023. “Glutamate Decarboxylase‐1 Is Essential for Efficient Acclimation of *Arabidopsis thaliana* to Nutritional Phosphorus Deprivation.” New Phytologist 240, no. 6: 2372–2385. 10.1111/nph.19300. 37837235

[fsn372125-bib-0005] Bhattacharjee, P. , S. Chakraborti , S. Chakraborty , and K. Paul . 2018. “The Role of Gamma Aminobutyric Acid (GABA) During Abiotic Stress in Plants.” In Metabolic Adaptations in Plants During Abiotic Stress. CRC Press.

[fsn372125-bib-0006] Bown, A. W. , and B. J. Shelp . 1997. “The Metabolism and Functions of [Gamma]‐Aminobutyric Acid.” Plant Physiology 115, no. 1: 1–5. 10.1104/pp.115.1.1. 12223787 PMC158453

[fsn372125-bib-0007] Cao, B. , Z. Pan , Z. Nima , et al. 2010. “Distribution of γ‐Aminobutytric Acid in the Grains of Naked Barley Collected From Qinghai‐Tibet Plateau and Abroad.” Journal of Triticeae Crops 30, no. 3: 555–559. https://kns.cnki.net/KCMS/detail/detail.aspx?dbcode=CJFQ&dbname=CJFD2010&filename=MLZW201003036.

[fsn372125-bib-0008] Chen, F. , Y. Wang , Y. Liu , et al. 2025. “Exogenous γ‐Aminobutyric Acid (GABA) Provides a Carbon Skeleton to Promote the Accumulation of Sugar and Unsaturated Fatty Acids in Vegetable Soybean Seeds.” Environmental and Experimental Botany 229: 106052. 10.1016/j.envexpbot.2024.106052.

[fsn372125-bib-0009] Chen, L. , B. Liu , S. Feng , X. Ma , S. Wang , and Y. Zhang . 2023. “Correlation Between Microbe, Physicochemical Properties of Jiuqu in Different Plateau Areas and Volatile Flavor Compounds of Highland Barley Alcoholic Drink.” Food Bioscience 51: 102276. 10.1016/j.fbio.2022.102276.

[fsn372125-bib-0010] Du, Y. , Z. Chen , F. Liang , et al. 2022. “Effects of Hypoxia Stress Germination on Nutrients, Physicochemical Properties and Cooking Characteristics of Highland Barley.” Journal of Cereal Science 103: 103411. 10.1016/j.jcs.2021.103411.

[fsn372125-bib-0011] Fait, A. , H. Fromm , D. Walter , G. Galili , and A. R. Fernie . 2008. “Highway or Byway: The Metabolic Role of the GABA Shunt in Plants.” Trends in Plant Science 13, no. 1: 14–19. 10.1016/j.tplants.2007.10.005. 18155636

[fsn372125-bib-0012] Feehily, C. , and K. A. G. Karatzas . 2013. “Role of Glutamate Metabolism in Bacterial Responses Towards Acid and Other Stresses.” Journal of Applied Microbiology 114, no. 1: 11–24. 10.1111/j.1365-2672.2012.05434.x. 22924898

[fsn372125-bib-0013] Ge, S. , L. Han , S. Hou , et al. 2025. “Influence of Cooking Methods on Flavor Parameters and Sensory Quality of Tibetan Sheep Meat Examined Using an Electronic Nose, an Electronic Tongue, GC–IMS, and GC–MS.” Food 14, no. 13: 2181. 10.3390/foods14132181. PMC12248567 40646933

[fsn372125-bib-0014] Han, C. , S. Zhen , G. Zhu , Y. Bian , and Y. Yan . 2017. “Comparative Metabolome Analysis of Wheat Embryo and Endosperm Reveals the Dynamic Changes of Metabolites During Seed Germination.” Plant Physiology and Biochemistry 115: 320–327. 10.1016/j.plaphy.2017.04.013. 28415032

[fsn372125-bib-0015] Hu, Y. , X. Huang , Q. Xiao , et al. 2024. “Advances in Plant GABA Research: Biological Functions, Synthesis Mechanisms and Regulatory Pathways.” Plants 13, no. 20: 2891. 10.3390/plants13202891. 39458838 PMC11510998

[fsn372125-bib-0016] Huang, J. , H. Fang , Z.‐C. Gai , et al. 2018. “ *Lactobacillus brevis* CGMCC 1306 Glutamate Decarboxylase: Crystal Structure and Functional Analysis.” Biochemical and Biophysical Research Communications 503, no. 3: 1703–1709. 10.1016/j.bbrc.2018.07.102. 30049439

[fsn372125-bib-0017] Jiang, X. , Q. Xu , A. Zhang , et al. 2021. “Optimization of γ‐Aminobutyric Acid (GABA) Accumulation in Germinating Adzuki Beans ( *vigna angularis* ) by Vacuum Treatment and Monosodium Glutamate, and the Molecular Mechanisms.” Frontiers in Nutrition 8: 693862. 10.3389/fnut.2021.693862. 34568402 PMC8458712

[fsn372125-bib-0018] Jiang, X. , Q. Xu , J. Zhang , et al. 2023. “Nutrient Transfer and Antioxidant Effect of Adzuki Bean Before and After GABA Enrichment.” Frontiers in Nutrition 10: 1123075. 10.3389/fnut.2023.1123075. 36776599 PMC9909224

[fsn372125-bib-0019] Jin, Y. , J. Tu , X. Han , et al. 2022. “Characteristics of Mulberry Leaf Powder Enriched With γ‐Aminobutyric Acid and Its Antioxidant Capacity as a Potential Functional Food Ingredient.” Frontiers in Nutrition 9: 900718. 10.3389/fnut.2022.900718. 35662930 PMC9158535

[fsn372125-bib-0020] Karki, R. , P. Ojha , S. Maharjan , U. Manandhar , and S. Maharjan . 2025. “Optimization of the Germination Time of Proso and Foxtail Millets to Enhance the Bioactive Properties, Antioxidant Activity, and Enzymatic Power and Reduce Antinutritional Factor.” Current Research in Food Science 10: 100987. 10.1016/j.crfs.2025.100987. 40114744 PMC11923759

[fsn372125-bib-0021] Kumar, N. , A. Gautam , A. K. Dubey , et al. 2019. “GABA Mediated Reduction of Arsenite Toxicity in Rice Seedling Through Modulation of Fatty Acids, Stress Responsive Amino Acids and Polyamines Biosynthesis.” Ecotoxicology and Environmental Safety 173: 15–27. 10.1016/j.ecoenv.2019.02.017. 30743076

[fsn372125-bib-0022] Li, W. , Y. Liu , R. Tang , P. Liao , and L. Ma . 2019. “Preparation of γ‐Aminobutyric Acid Using Whole‐Cell Biotransformation.” Journal of Hubei University (Natural Science) 41, no. 1: 5–9. https://kns.cnki.net/KCMS/detail/detail.aspx?dbcode=CJFQ&dbname=CJFDLAST2019&filename=HDZK201901002.

[fsn372125-bib-0023] Liu, S. , W. Wang , H. Lu , Q. Shu , Y. Zhang , and Q. Chen . 2022. “New Perspectives on Physiological, Biochemical and Bioactive Components During Germination of Edible Seeds: A Review.” Trends in Food Science & Technology 123: 187–197. 10.1016/j.tifs.2022.02.029.

[fsn372125-bib-0024] Ma, J. , J. Du , W. Zhang , et al. 2025. “Analysis of Flavor Metabolism Characteristics of Quinoa ( *Chenopodium quinoa* Willd.) Sprout Plant Beverage by GC‐IMS Combined With Intelligent Sensory Technology.” Food Research International 221: 117373. 10.1016/j.foodres.2025.117373. 41174446

[fsn372125-bib-0025] Minchán‐Velayarce, H. H. , A.‐S. Bustos , L. M. Paucar‐Menacho , J. Vidaurre‐Ruiz , and M. Schmiele . 2025. “New Frontiers in Cereal and Pseudocereal Germination: Emerging Inducers for Maximizing Bioactive Compounds.” Food 14, no. 17: 3090. 10.3390/foods14173090. PMC12428404 40941206

[fsn372125-bib-0026] Obadi, M. , J. Sun , and B. Xu . 2021. “Highland Barley: Chemical Composition, Bioactive Compounds, Health Effects, and Applications.” Food Research International 140: 110065. 10.1016/j.foodres.2020.110065. 33648288

[fsn372125-bib-0027] Oh, S.‐J. , H. S. Kim , S.‐T. Lim , and C. K. Reddy . 2019. “Enhanced Accumulation of Gamma‐Aminobutyric Acid in Rice Bran Using Anaerobic Incubation With Various Additives.” Food Chemistry 271: 187–192. 10.1016/j.foodchem.2018.07.175. 30236666

[fsn372125-bib-0028] Palabıyık, Ş. , İ. Çetinkaya , T. A. Öztürk , and M. Bor . 2024. “Flagellin Induced GABA‐Shunt Improves Drought Stress Tolerance in *brassica napus* L.” BMC Plant Biology 24, no. 1: 864. 10.1186/s12870-024-05503-9. 39278927 PMC11403839

[fsn372125-bib-0029] Peng, L. , L. Hertz , R. Huang , et al. 1994. “Utilization of Glutamine and of TCA Cycle Constituents as Precursors for Transmitter Glutamate and GABA.” Developmental Neuroscience 15, no. 3–5: 367–377. 10.1159/000111357. 7805591

[fsn372125-bib-0030] Qu, C. , H. Zhao , J. Chen , et al. 2019. “The Transcriptional Events and Their Relationship to Physiological Changes During Poplar Seed Germination and Post‐Germination.” BMC Genomics 20, no. 1: 801. 10.1186/s12864-019-6180-5. 31684868 PMC6829952

[fsn372125-bib-0031] Rideaux, R. , S. E. Ehrhardt , Y. Wards , et al. 2022. “On the Relationship Between GABA+ and Glutamate Across the Brain.” NeuroImage 257: 119273. 10.1016/j.neuroimage.2022.119273. 35526748 PMC9924060

[fsn372125-bib-0032] Salaberría, F. , S. Marzocchi , E. Bortolazzo , M. E. Carrín , and M. F. Caboni . 2021. “Study of the Effect of NaCl on Lipolysis in Parmigiano Reggiano Cheese.” ACS Food Science & Technology 1, no. 1: 54–59. 10.1021/acsfoodscitech.0c00025.

[fsn372125-bib-0033] Sarasa, S. B. , M. Ramasamy , M. Gayathri , T. Bency , S. D. R. Femil , and A. Jayaraman . 2020. “A Brief Review on the Non‐Protein Amino Acid, Gamma‐Amino Butyric Acid (GABA): Its Production and Role in Microbes.” Current Microbiology 77, no. 4: 534–544. 10.1007/s00284-019-01839-w. 31844936

[fsn372125-bib-0034] Schmidt, C. , R. Seibel , M. Wehsling , et al. 2020. “Keto Leucine and Keto Isoleucine Are Bioavailable Precursors of Their Respective Amino Acids in Cell Culture Media.” Journal of Biotechnology 321: 1–12. 10.1016/j.jbiotec.2020.06.013. 32580011

[fsn372125-bib-0035] Sharma, S. , D. C. Saxena , and C. S. Riar . 2017. “Using Combined Optimization, GC–MS and Analytical Technique to Analyze the Germination Effect on Phenolics, Dietary Fibers, Minerals and GABA Contents of Kodo Millet ( *Paspalum scrobiculatum* ).” Food Chemistry 233: 20–28. 10.1016/j.foodchem.2017.04.099. 28530567

[fsn372125-bib-0036] Shen, L. , Y. Yang , X. Liu , et al. 2025. “Strategies to Improve γ‐Aminobutyric Acid Biosynthesis in Rice via Optimal Conditions.” Plants 14, no. 9: 1290. 10.3390/plants14091290. 40364319 PMC12073630

[fsn372125-bib-0037] Sun, S. , J. Zhang , Y. Li , et al. 2024. “Effects of Sodium Selenite on Accumulations of Selenium and GABA, Phenolic Profiles, and Antioxidant Activity of Foxtail Millet During Germination.” Food 13, no. 23: 3916. 10.3390/foods13233916. PMC11641185 39682988

[fsn372125-bib-0038] Sun, Y. , A. Mehmood , M. Battino , J. Xiao , and X. Chen . 2022. “Enrichment of Gamma‐Aminobutyric Acid in Foods: From Conventional Methods to Innovative Technologies.” Food Research International 162: 111801. 10.1016/j.foodres.2022.111801. 36461174

[fsn372125-bib-0039] Tufail, T. , H. B. U. Ain , M. S. Virk , et al. 2025. “GABA (γ‐Aminobutyric Acid) Enrichment and Detection Methods in Cereals: Unlocking Sustainable Health Benefits.” Food Chemistry 464: 141750. 10.1016/j.foodchem.2024.141750. 39504899

[fsn372125-bib-0040] Utama, G. L. , N. R. M. Sahab , S. Nurmilah , V. P. Yarlina , E. Subroto , and R. L. Balia . 2025. “Unveiling Microbial Dynamics in Terasi Spontaneous Fermentation: Insights Into Glutamate and GABA Production.” Current Research in Food Science 10: 100950. 10.1016/j.crfs.2024.100950. 39760015 PMC11699049

[fsn372125-bib-0041] Wang, J. , J. Wang , Y. Gao , et al. 2025a. “Decoding Qingke's Aroma Compounds: Flavor Contributions, Interactions and Barley Contrast.” Food Chemistry 496: 146612. 10.1016/j.foodchem.2025.146612. 41109048

[fsn372125-bib-0042] Wang, J. , R. Wang , Y. Gao , et al. 2025b. “Analysis of Dynamic Flavor Changes of Volatile and Non‐Volatile Fractions Analysis of Black Qingke ( *Hordeum vulgare* L. Var. Nudum Hook. F.) During Steaming Process.” Journal of Food Science 90, no. 2: e17660. 10.1111/1750-3841.17660. 39929602

[fsn372125-bib-0043] Wang, J. , R. Wang , J. Wang , Y. Gao , L. Qiao , and N. Zhang . 2025. “Recognition of Characteristic Aroma‐Active Components in Qingke (Highand Barley) Through Flavoromics Analysis.” Food Chemistry 492: 145262. 10.1016/j.foodchem.2025.145262. 40602137

[fsn372125-bib-0044] Wang, Y. , Y. He , Y. Liu , and D. Wang . 2023. “Analyzing Volatile Compounds of Young and Mature Docynia Delavayi Fruit by HS‐SPME‐GC‐MS and rOAV.” Food 12, no. 1: 59. 10.3390/foods12010059. PMC9818226 36613274

[fsn372125-bib-0045] Wu, S. , B. Chen , X. Wang , et al. 2025. “Functional Properties and Flavor of Fermented Germinated Brown Rice‐Chickpea Beverage With *lactobacillus plantarum* .” Food Chemistry: X 32: 103231. 10.1016/j.fochx.2025.103231. 41282316 PMC12639554

[fsn372125-bib-0046] Wu, X. , Q. Jia , S. Ji , et al. 2020. “Gamma‐Aminobutyric Acid (GABA) Alleviates Salt Damage in Tomato by Modulating Na+ Uptake, the GAD Gene, Amino Acid Synthesis and Reactive Oxygen Species Metabolism.” BMC Plant Biology 20, no. 1: 465. 10.1186/s12870-020-02669-w. 33036565 PMC7547442

[fsn372125-bib-0047] Xie, C. , M. Sun , P. Wang , and R. Yang . 2022. “Interaction of Gamma‐Aminobutyric Acid and Ca2+ on Phenolic Compounds Bioaccumulation in Soybean Sprouts Under NaCl Stress.” Plants 11, no. 24: 3503. 10.3390/plants11243503. 36559615 PMC9787623

[fsn372125-bib-0048] Yamamoto, T. , and C. Inui‐Yamamoto . 2023. “The Flavor‐Enhancing Action of Glutamate and Its Mechanism Involving the Notion of Kokumi.” npj Science of Food 7, no. 1: 3. 10.1038/s41538-023-00178-2. 36707516 PMC9883458

[fsn372125-bib-0049] Yin, Z. , R. Yan , Y. Jiang , et al. 2022. “Identification of Peptides in Qingke Baijiu and Evaluation of Its Angiotensin Converting Enzyme (ACE) Inhibitory Activity and Stability.” Food Chemistry 395: 133551. 10.1016/j.foodchem.2022.133551. 35802984

[fsn372125-bib-0050] Youssef, H. A. I. , P. Vitaglione , R. Ferracane , J. Abuqwider , and G. Mauriello . 2023. “Evaluation of GABA Production by Alginate‐Microencapsulated Fresh and Freeze‐Dried Bacteria Enriched With Monosodium Glutamate During Storage in Chocolate Milk.” Microorganisms 11, no. 11: 2648. 10.3390/microorganisms11112648. 38004660 PMC10673371

[fsn372125-bib-0051] Zhang, D. , X. Wei , Z. Liu , et al. 2021. “Transcriptome Analysis Reveals the Molecular Mechanism of GABA Accumulation During Quinoa ( *Chenopodium quinoa* willd.) Germination.” Journal of Agricultural and Food Chemistry 69, no. 41: 12171–12186. 10.1021/acs.jafc.1c02933. 34610747

[fsn372125-bib-0052] Zhang, W. , X. Yang , J. Zhang , Y. Lan , and B. Dang . 2023. “Study on the Changes in Volatile Flavor Compounds in Whole Highland Barley Flour During Accelerated Storage After Different Processing Methods.” Food 12, no. 11: 2137. 10.3390/foods12112137. PMC10252761 37297381

[fsn372125-bib-0053] Zhang, X. , L. Han , S. Hou , et al. 2022. “Effects of Different Feeding Regimes on Muscle Metabolism and Its Association With Meat Quality of Tibetan Sheep.” Food Chemistry 374: 131611. 10.1016/j.foodchem.2021.131611. 34863603

[fsn372125-bib-0054] Zhou, M. , M. J. Hassan , Y. Peng , et al. 2021. “γ‐Aminobutyric Acid (GABA) Priming Improves Seed Germination and Seedling Stress Tolerance Associated With Enhanced Antioxidant Metabolism, DREB Expression, and Dehydrin Accumulation in White Clover Under Water Stress.” Frontiers in Plant Science 12: 776939. 10.3389/fpls.2021.776939. 34925419 PMC8678086

[fsn372125-bib-0055] Zhu, Y. , S. Tan , C. Xie , et al. 2024. “Effects of Magnetic Field Pretreatment and Chloride Salt Stress on Physio‐Biochemical Changes and γ‐Aminobutyric Acid Accumulation in Germinated Brown Rice.” Food Materials Research 4, no. 1: e015. 10.48130/fmr-0024-0006.

