# Supplementary material for: Effects of Exogenous Monosodium Glutamate on GABA Accumulation and Volatile/Nonvolatile Metabolites in Black Highland Barley
Source: Food Sci Nutr. 2026 Jul 29;14(8):e72125. doi: 10.1002/fsn3.72125 (PMC13416752; doi:10.1002/fsn3.72125)
Supplement: Supplementary file 1 — Figure S1: Overlaid total ion chromatograms of QC samples in positive (A) and negative (B) ion modes. Table S1: Differential metabolites identified in Glu‐0, Glu‐50, and Glu‐75 groups. [file FSN3-14-e72125-s001.zip › Supplementary Figure 1.docx]

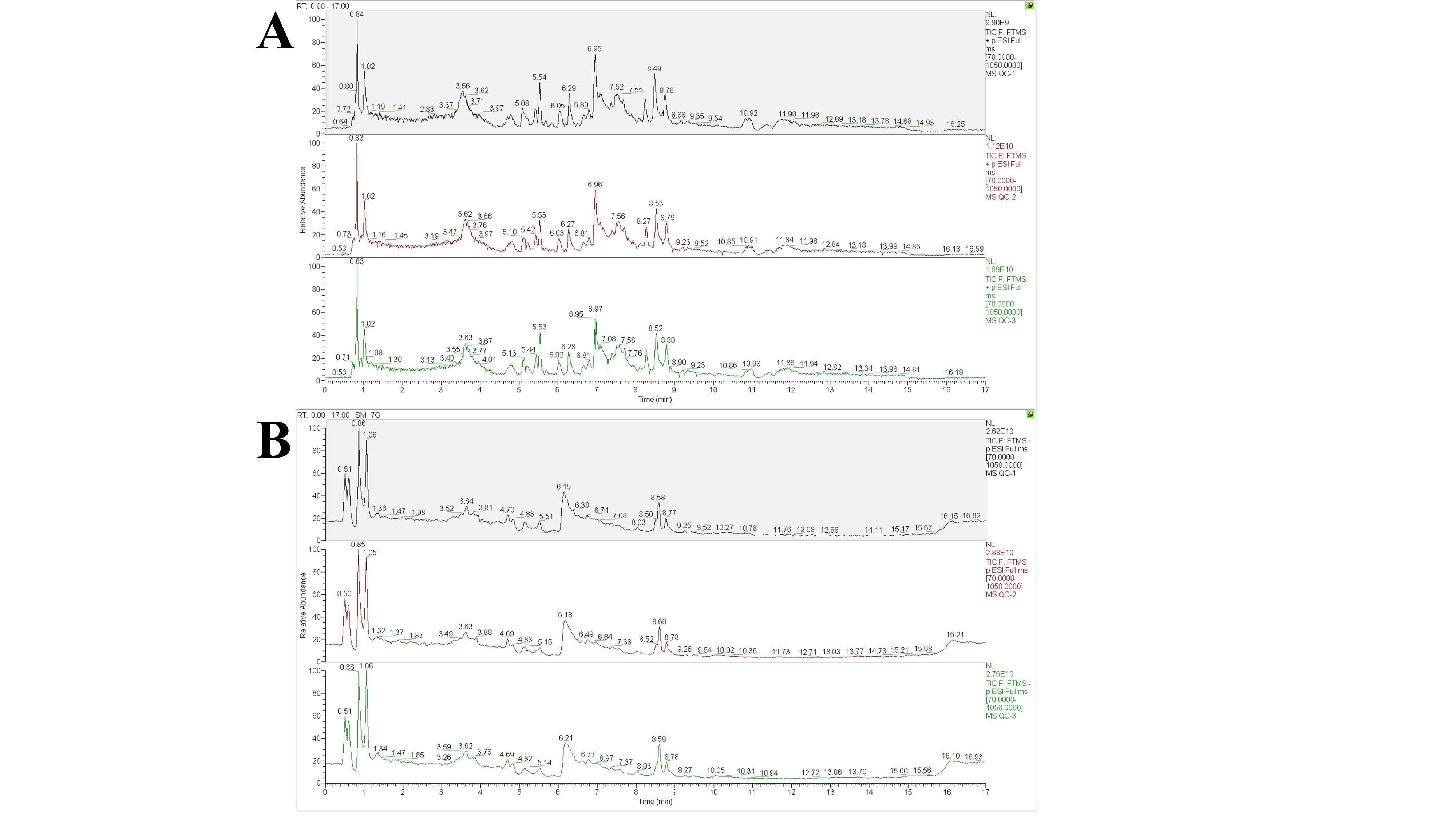


Supplementary Fig 1. Overlaid total ion chromatograms of QC samples in positive (A) and negative (B) ion modes.
